# Microfluidics-free single-cell genomics with templated emulsification

**DOI:** 10.1038/s41587-023-01685-z

**Published:** 2023-03-06

**Authors:** Iain C. Clark, Kristina M. Fontanez, Robert H. Meltzer, Yi Xue, Corey Hayford, Aaron May-Zhang, Chris D’Amato, Ahmad Osman, Jesse Q. Zhang, Pabodha Hettige, Jacob S. A. Ishibashi, Cyrille L. Delley, Daniel W. Weisgerber, Joseph M. Replogle, Marco Jost, Kiet T. Phong, Vanessa E. Kennedy, Cheryl A. C. Peretz, Esther A. Kim, Siyou Song, William Karlon, Jonathan S. Weissman, Catherine C. Smith, Zev J. Gartner, Adam R. Abate

**Affiliations:** 1 Department of Bioengineering, University of California, Berkeley, California Institute for Quantitative Biosciences, Berkeley, CA USA; 2 Fluent Biosciences, Watertown, MA USA; 3 Department of Bioengineering and Therapeutic Sciences, University of California San Francisco, San Francisco, CA USA; 4 Whitehead Institute for Biomedical Research, Massachusetts Institute of Technology, Cambridge, MA USA; 5 Department of Pharmaceutical Chemistry, University of California San Francisco, San Francisco, CA USA; 6 Department of Medicine, University of California San Francisco, San Francisco, CA USA; 7 Department of Pediatrics, University of California San Francisco, San Francisco, CA USA; 8 Division of Plastic and Reconstructive Surgery, University of California San Francisco, San Francisco, CA USA; 9 Departments of Pathology and Laboratory Medicine, University of California San Francisco, San Francisco, CA USA; 10 Howard Hughes Medical Institute, Massachusetts Institute of Technology, Cambridge, MA USA; 11 Chan Zuckerberg Biohub, San Francisco, CA USA; 12 Present Address: Department of Microbiology, Harvard Medical School, Boston, MA USA

**Keywords:** Gene expression analysis, Sequencing, Functional genomics

## Abstract

Current single-cell RNA-sequencing approaches have limitations that stem from the microfluidic devices or fluid handling steps required for sample processing. We develop a method that does not require specialized microfluidic devices, expertise or hardware. Our approach is based on particle-templated emulsification, which allows single-cell encapsulation and barcoding of cDNA in uniform droplet emulsions with only a vortexer. Particle-templated instant partition sequencing (PIP-seq) accommodates a wide range of emulsification formats, including microwell plates and large-volume conical tubes, enabling thousands of samples or millions of cells to be processed in minutes. We demonstrate that PIP-seq produces high-purity transcriptomes in mouse–human mixing studies, is compatible with multiomics measurements and can accurately characterize cell types in human breast tissue compared to a commercial microfluidic platform. Single-cell transcriptional profiling of mixed phenotype acute leukemia using PIP-seq reveals the emergence of heterogeneity within chemotherapy-resistant cell subsets that were hidden by standard immunophenotyping. PIP-seq is a simple, flexible and scalable next-generation workflow that extends single-cell sequencing to new applications.

## Abstract (web summary)

A microfluidics-free, scalable single-cell RNA-sequencing method produces high-quality transcriptomes.

## Main

Single-cell RNA sequencing (scRNA-seq) is an essential technology in the biological sciences because it reveals how the properties of tissues arise from the transcriptional states of numerous interacting cells. Defining the gene expression signatures of individual cells allows cell-type classification, the discovery of unique cell states during development and disease and the prediction of regulatory mechanisms that control these states. As a result, bulk sequencing is being rapidly replaced by single-cell methods. The first single-cell approaches isolated cells and prepared them individually for sequencing^1–4^. While improvements in molecular biology increased data quality^5,6^, the requisite isolation and processing of separate cells ultimately limited throughput. Implementation of valve-based microfluidics reduced hands-on time^7^ but failed to substantially increase cell number and thus could not capture the heterogeneity intrinsic to most tissues. Advances in high-throughput droplet microfluidic barcoding have expanded single-cell sequencing to tens of thousands of cells^8,9^ and fueled biological discovery but require expensive instruments located in core facilities and therefore remain inaccessible to many labs. Methods for direct combinatorial indexing of cells^10,11^, the use of nanowell arrays^12^ or sample multiplexing^13,14^ have overcome some limitations of microfluidics, but no current method simultaneously accommodates both low (10) and high (>10^6^) cell numbers, can be applied to hundreds of independent samples and can be rapidly implemented without custom equipment.

The scalability of single-cell methods is important for many applications, including tissue atlas projects^15–18^, million-cell perturbation experiments^19^, drug development pipelines^20^ and developmental studies^21^. Droplet microfluidics has an intrinsic disadvantage at high cell numbers due to the upper limit on drop generation speed. At high fluid velocities, droplet generation becomes uncontrolled, resulting in polydispersed emulsions and poor bead loading that reduces single-cell data quality^22,23^. Therefore, to sequence millions of cells requires long run times, parallel droplet generators with complex designs that are prone to clogging or implementation of additional barcoding steps before encapsulation^24^. More generally, droplet microfluidics relies on an expensive instrument usually located in a core facility, which necessitates sample transport or fixation that can alter RNA profiles. Centralized processing also reduces access to many labs and does not fit experiments that need rapid or point-of-collection sample handling, such as remote fieldwork or studies using infectious samples requiring biosafety precautions^12,25^.

Much effort has thus gone into developing microfluidics-free single-cell methods. Split-pool ligation^10,11^ and tagmentation^26,27^ perform direct combinatorial barcoding of bulk suspensions and substantially increase cell number; however, these laborious workflows require enormous numbers of pipetting operations and are poorly suited for low cell inputs. Moreover, while scalable, these methods require substantial expertise^28^, and broad adoption of split-pool barcoding will likely require robotic automation in a centralized facility. Alternatively, methods based on nanowells prioritize simplicity and cost-effectiveness^12,25^. No microfluidics are required, and wells are loaded by sedimentation, providing an instrument-free and point-of-use solution. However, nanowell array chips do not efficiently scale in cell or sample number; the planar arrays capture cells on a two-dimensional surface and, thus, cannot compete with emulsions or combinatorial indexing using a three-dimensional volume that easily scales to millions of cells. Moreover, unless combined with multiplexing^13,14^, nanowell chips are poorly suited for processing many separate samples because they require one array per sample and thus hundreds of arrays for hundreds of samples. To advance the field of single-cell genomics, next-generation technologies must simultaneously innovate on speed, scale and ease of use. An ideal system would be compatible with the barcoding of separate samples in well plates, accommodate orders-of-magnitude differences in cell number, be completed in minutes and be easy to run at the bench or in the field without specialized instrumentation.

Here, we describe a flexible, scalable and instrument-free scRNA-seq method based on rapid templated emulsification of cells and barcoded hydrogel templates without microfluidics^29^. In contrast to microfluidic emulsification, in which droplets are created sequentially and thus their number scales with instrument run time, templated emulsification generates monodispersed droplets in parallel by bulk self-assembly, and, thus, the number of droplets (and cells that can be barcoded) scales only with container volume. The result is a scalable, user-friendly scRNA-seq method that we call particle-templated instant partition sequencing (PIP-seq). Templated emulsification produces drops that are equivalent to those generated with microfluidics and compatible with the latest innovations in multiomic measurements. Here, we show that PIP-seq generates accurate single-cell gene expression profiles from human tissues and is compatible with multimodal measurements of RNA and single guide RNA (sgRNA; CRISPR droplet sequencing (CROP-seq)) or RNA and protein (cellular indexing of transcriptomes and epitopes sequencing (CITE-seq)). Finally, we demonstrate the use of PIP-seq to monitor the response of individuals with mixed phenotype acute leukemia (MPAL) to chemotherapy, revealing heterogeneity within cells with similar immunophenotypes. In summary, PIP-seq fills an unmet technical need by improving the speed, scalability and ease of use of single-cell sequencing.

## Results

### Overview of the technology

**Fig. 1:**
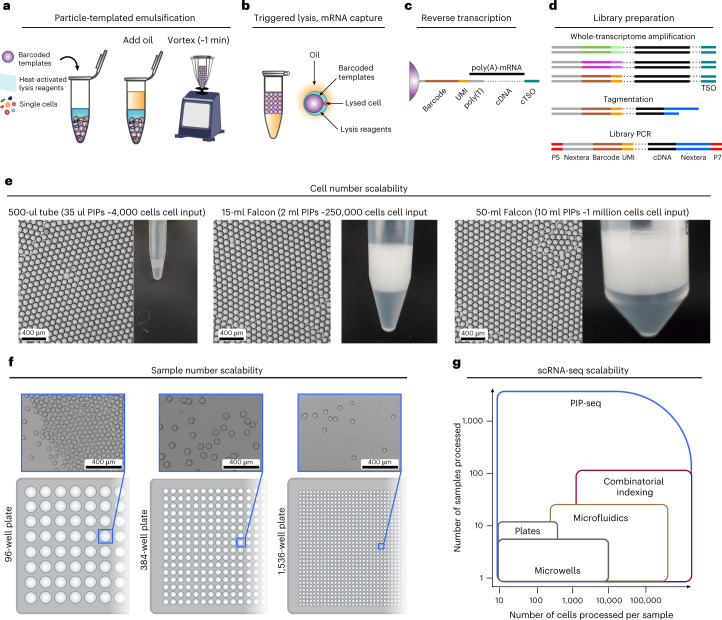
Rapid and scalable templated emulsification for single-cell genomics. **a**–**d**, PIP-seq enables the encapsulation, lysis and barcoding of single cells. **a**, Schematic of the emulsification process. Barcoded particle templates, cells and lysis reagents are combined with oil and vortexed to generate monodispersed droplets. **b**, Heat activation of PK results in lysis and release of mRNA that is captured on bead-bound barcoded poly(T) oligonucleotides. **c**, Oil removal is followed by bulk reverse transcription of mRNA into cDNA. cTSO is the complement of the template switch oligonucleotide. **d**, Barcoded whole-transcriptome-amplified cDNA is prepared for Illumina sequencing. **e**–**g**, Efficient single-bead, single-drop encapsulation at scale. **e**, Particle-templated emulsification in different-sized tubes (1.5 ml, 15 ml and 50 ml) produces monodispersed emulsions capable of barcoding orders of magnitude different cell numbers. **f**, PIP-seq is compatible with plate-based emulsification, including 96-, 384- and 1,536-well plate formats. Representative images are shown from experiments completed three times. **g**, The estimated ability of different technologies to easily scale with respect to cell and sample number.

**Extended Data Fig. 1:**
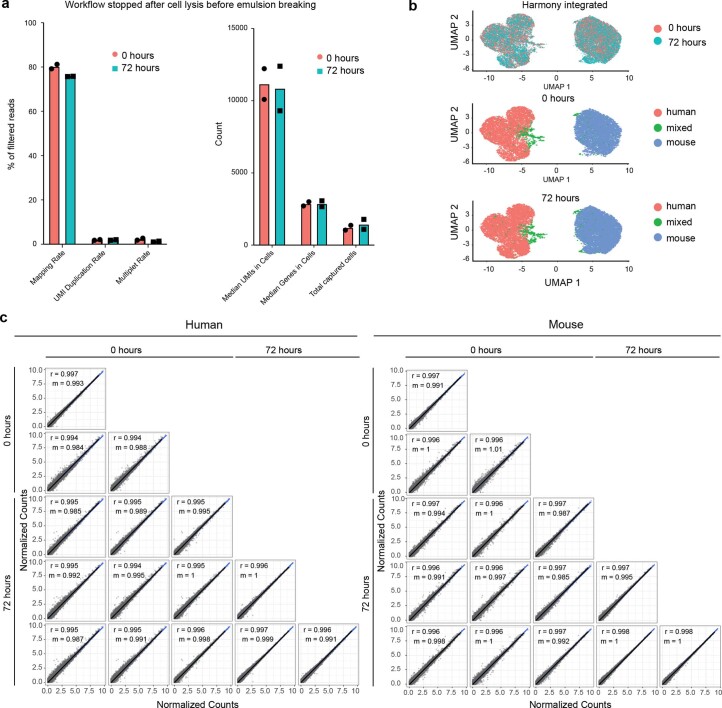
Flexible sample processing with PIP-seq. **(a)** Storage of droplets after emulsification for 72 hours at 0 °C did not change quality metrics. Each bar represents the average of two biologically independent experiments with the individual data points shown. **(b)** Data integration between time points. **(c)** Correlations in normalized gene expression, by sample, between time points for mouse and human cells.

PIP-seq uses particle templating to compartmentalize cells, barcoded hydrogel templates and lysis reagents in monodispersed water-in-oil droplets (Fig. 1a). Rapid emulsification with a standard vortexer allows cells to be encapsulated at the bench or point of collection in minutes. The cells are lysed by increasing the temperature to 65 °C, which activates proteinase K (PK), releasing cellular mRNA that is captured on polyacrylamide beads decorated with barcoded poly(T) sequences (Fig. 1b). PIP-seq emulsions can be stored for days at 0 °C without change in data quality (Extended Data Fig. 1), allowing samples to be banked for future processing. After resuming, oil is removed, beads are transferred into a reverse transcription buffer, and full-length cDNA is synthesized, amplified and prepared for sequencing (Fig. 1c,d).

**Extended Data Fig. 2:**
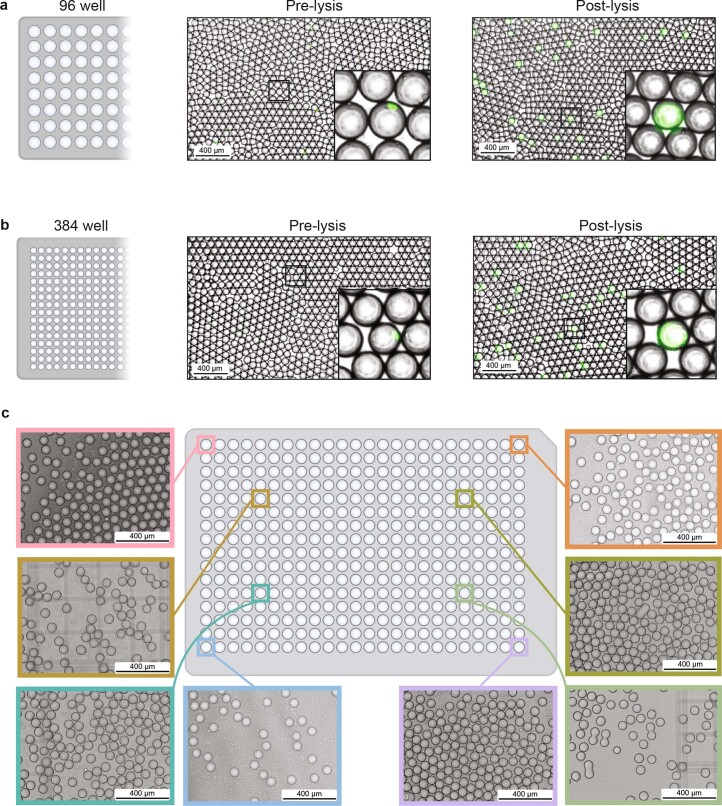
High cell and sample number experiments with PIP-seq. **(a-c)** Microscope images of droplets and cells in plate emulsification experiments. Representative images are from experiments completed at least three times. **(a,b)** Barcode bead templates, stained cells (puncta), and lysis reagents are combined with oil and vortexed in **(a)** 96-well and **(b)** 384-well plates to generate monodispersed droplets. Heat activation of Proteinase K results in lysis and release of calcein dye, and full-drop fluorescence. **(c)** Microscope images of droplets from random wells of a 384-well plate emulsification experiment.

A unique and valuable feature of PIP-seq is that cell encapsulation in droplets is performed in parallel using bead size to control droplet volume. In contrast to microfluidics, the number of droplets scales with total container volume, not emulsification time. For example, at a 6% collision rate that includes cell doublets and barcode reuse, we estimate that 3,500 cells can be barcoded with 35 µl of barcoded hydrogel templates in a 500-µl tube, 225,000 cells can be barcoded with 2 ml of barcoded hydrogel templates in a 15-ml conical tube, and 1 million cells can be barcoded with 10 ml of barcoded hydrogel templates in a 50-ml conical tube (Fig. 1e). Regardless of the tube size, only 2 min of vortexing is required for cell capture. PIP-seq is equally scalable to large sample numbers. Encapsulation can be performed directly in 96-, 384- or 1,536-well plates (Fig. 1f and Extended Data Fig. 2), greatly simplifying experiments testing hundreds of different conditions and streamlining integration with robotic handling systems. Thus, compared to current scRNA-seq technologies, PIP-seq has the greatest flexibility to cover combinations of cell and sample numbers (Fig. 1g).

### scRNA-seq with particle-templated emulsification

**Fig. 2:**
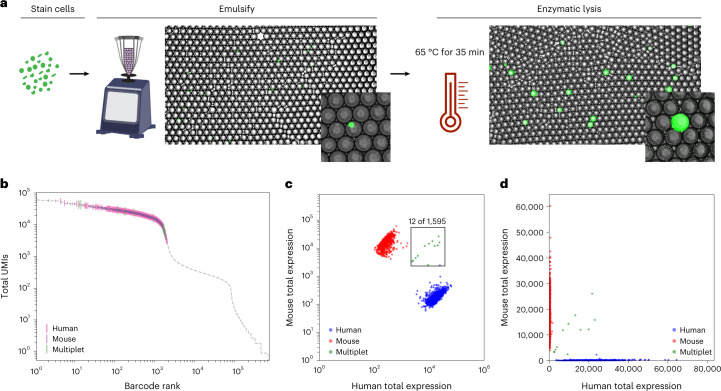
Heat-activated enzymatic lysis yields high-purity single-cell transcriptomes. **a**, Fluorescence microscopy (brightfield and green fluorescent protein) of calcein-stained cells emulsified with barcoded bead templates before and after heat-activated lysis. Inset images show cell puncta (left) and release of calcein (right) after lysis. Representative images are shown from experiments completed at least three times. **b**–**d**, Cell purity assessed with mouse–human mixing studies. **b**, Distribution of total UMIs as a function of cell barcode rank. The gray line represents all barcode groups, with called cells colored by species. **c**,**d**, Purity analysis of cell transcriptomes assessed using barnyard plots. Cells are colored by cell type (red, mouse reads; blue, human reads; green, mixed reads). Representative data are shown from species-mixing experiments completed over ten times.

High-throughput single-cell sequencing requires efficient cell lysis and reverse transcription of mRNA using barcoded primers. In the absence of microfluidics, barcoded hydrogel templates, cells and lysis reagents must be combined before emulsification. To prevent cell lysis before compartmentalization, we use PK, a protease that has minimal activity at 4 °C but can be activated at higher temperatures. After emulsification, the sample is heated to efficiently lyse cells. To illustrate this process, we stained cells with calcein, performed templated emulsification at 4 °C with PK and imaged the droplets before and after thermal activation. Intact cells appeared as compact puncta before lysis but rapidly released calcein into the bulk of the droplets after the temperature was increased (Fig. 2a and Extended Data Fig. 2a,b). Thus, cells can be mixed with PK in bulk before emulsification, and thermal activation triggers the release of mRNA for barcoding after emulsification.

To ensure that temperature-activated lysis and bulk agitation do not prelyse cells and result in mRNA cross-contamination, we performed mouse–human cell line mixing studies. We synthesized barcoded polyacrylamide beads with poly(T) sequences by using split-pool ligation of four 6-base pair (bp) randomers^30^. Beads contained ~10^8^ (96^4^) unique barcodes, providing ample sequence space to label 1 million cells. PIP-seq barcode rank plots for mixed mouse–human cell suspensions allowed cell identification by unique molecular identifier (UMI) abundance (Fig. 2b). The fraction of mouse reads in human transcriptomes was below 3%, and transcriptomes containing multiple cells were rare and consistent with Poisson encapsulation of two cells (Fig. 2c,d). These results illustrate that PIP-seq yields high-purity scRNA-seq data with minimal transcriptome mixing and low doublet formation.

### Accurate and scalable reconstruction of single-cell phenotypes in complex tissue

**Fig. 3:**
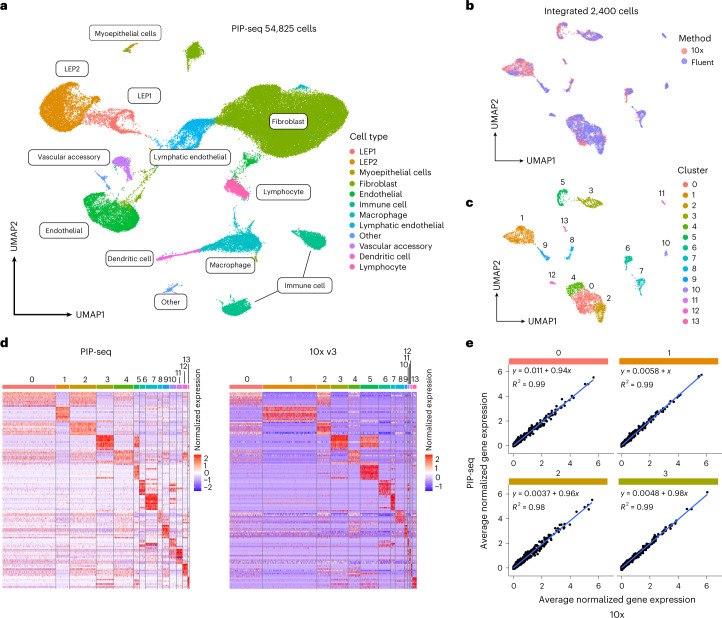
Accurate single-cell transcriptional profiling of healthy breast tissue using PIP-seq. **a**, Clustering and identification of cell types from PIP-seq data (54,825 cells from two individuals). **b**–**e**, Comparison of PIP-seq data to 10x Genomics data collected from the same tissue. **b**, Integration of PIP-seq and 10x data. **c**,**d**, Cell clustering and comparison of marker genes between platforms. **d**, Heat maps of marker gene expression show similar patterns in PIP-seq and 10x data. **e**, Correlations in normalized gene expression, by cluster, between platforms (see also Extended Data Fig. 4a).

**Extended Data Fig. 3:**
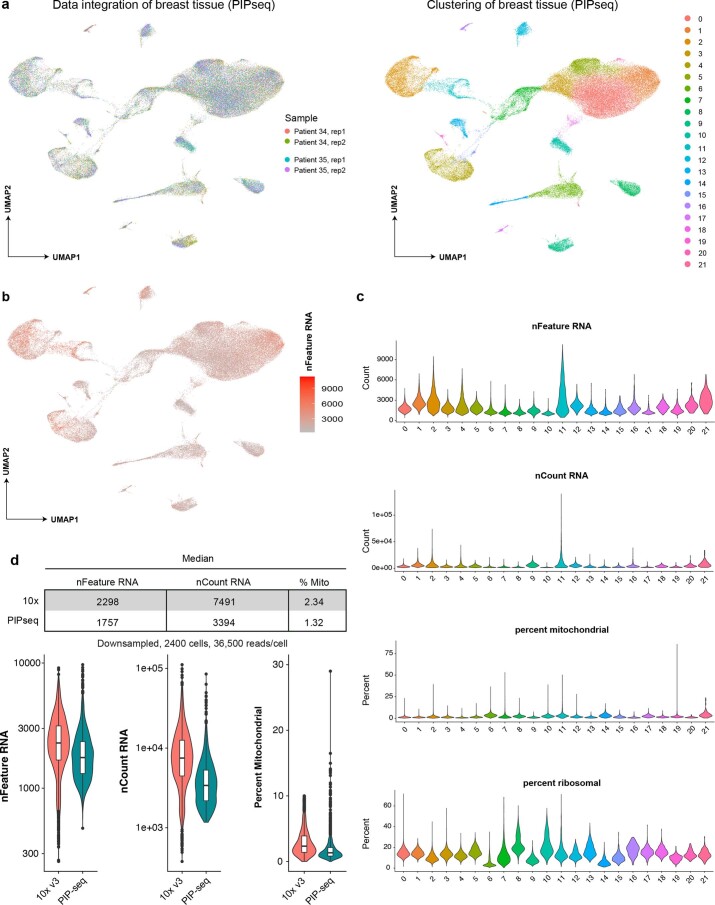
Quality control analysis of PIP-seq using healthy breast tissue. **(a)** Integration and clustering of 54,825 cells from 2 patients with 2 replicates per patient. **(b)** Coloring of UMAP by the number genes (nFeature RNA) for each cell. **(c)** The number of unique genes (nFeature RNA), transcripts (nCount RNA), percent mitochondrial reads, and percent ribosomal reads as a function of cluster. **(d)** Comparison between 10X Genomics’ and PIP-seq data after downsampling 2400 cells to 36,500 reads per cell. Box plots indicate the median with the lower and upper hinges corresponding to the 25th and 75th percentiles. **(c,d)** Each violin represents a combination of 4 individual samples (2 replicates from 2 patients).

**Extended Data Fig. 4:**
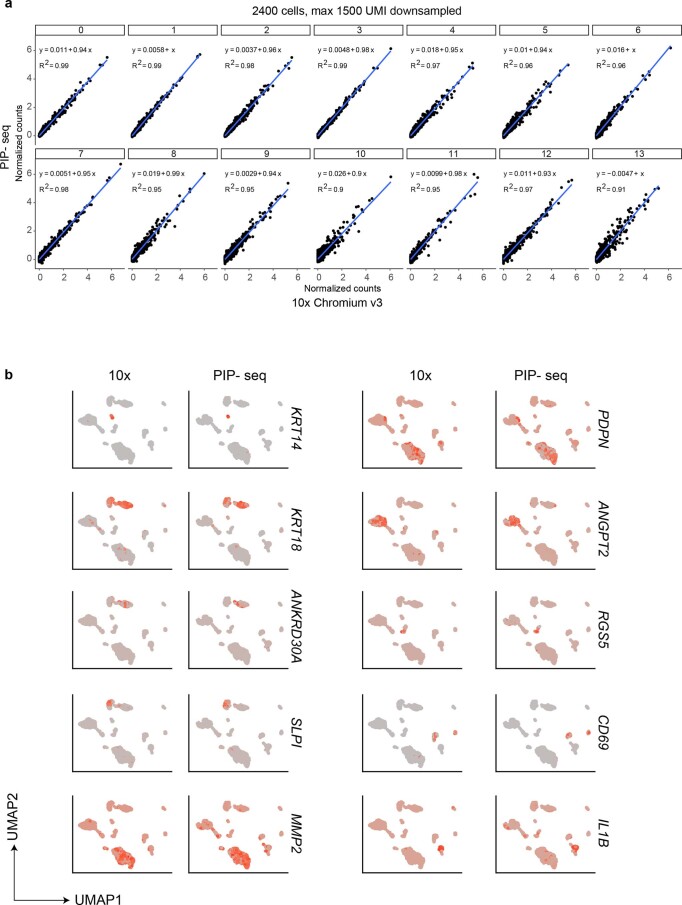
Comparison, after down-sampling (2400 cells 1500 UMIs) of the larger breast tissue PIP-seq dataset to 10x Genomics data generated from identical samples. **(a)** Correlations in normalized gene expression, by cluster, between platforms. **(b)** Expression of marker genes overlayed on clusters is consistent between platforms.

**Extended Data Fig. 5:**
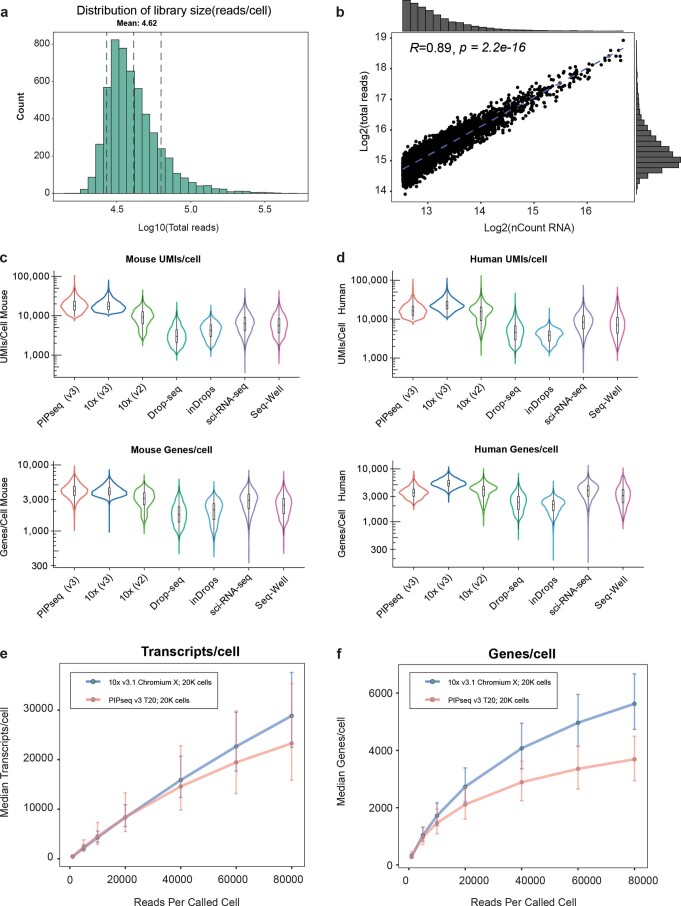
Data quality assessment of PIP-seq. **(a)** Representative distribution of reads per cell. **(b)** Correlation between reads and genes per cell. Spearman’s R and p values were calculated in R v4.1.0. **(c,d)** Comparison of UMIs/cell and genes/cell in current single-cell methods. Plots display a violin for a single representative sample for each platform. Transcripts per cell and genes per cell are separated by cell species (mouse (**c**) and human (**d**)), identified using the 85% species thresholding technique, as described in the methods. Box plots show the median, 25th and 75th percentiles. **(e,f)** Comparison of PIP-seq to 10X Genomics across a range of sequencing depths (0-80,000 reads/cell) (e) UMIs/cell and (f) genes/cell 80k cells down sampled from one biological replicate. Points represent the median with the lower and upper error bars corresponding to the 25th and 75th percentiles, respectively.

**Extended Data Fig. 6:**
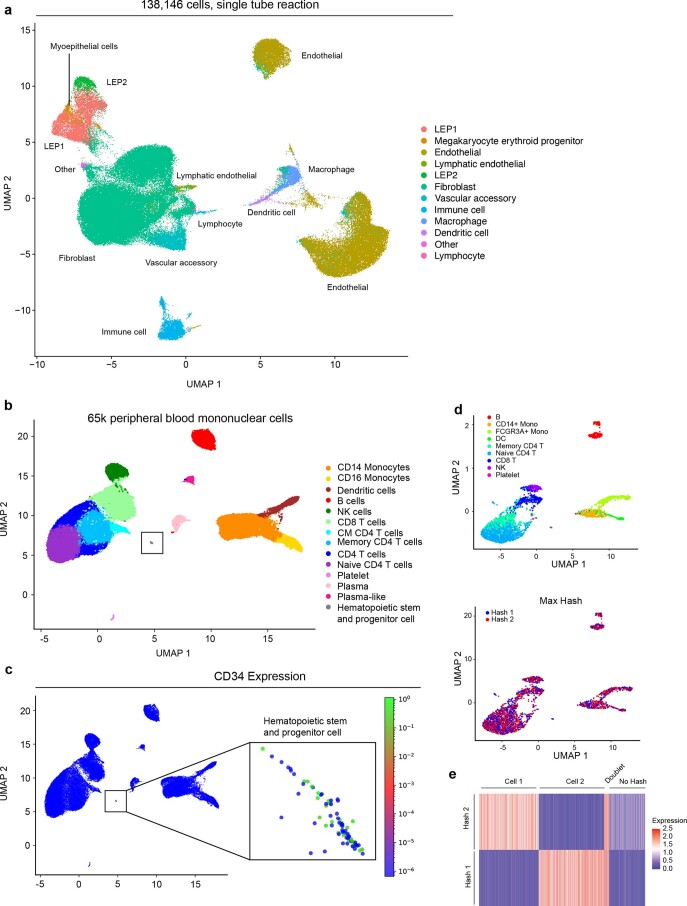
High cell number PIP-seq. **(a)** scRNA-seq of 138,146 single cells from breast tissue using a single-tube emulsification in 2-minutes. **(b)** scRNA-seq of 65k peripheral blood mononuclear cells (PBMCs) recovers a small population of CD34 cells. **(c)** Coloring of UMAP by the normalized expression of *CD34* for each cell. **(d,e)** Hashing of PBMCs demonstrates compatibility of PIP-seq with barcoded antibodies.

An important application of single-cell sequencing is atlasing cell types in heterogeneous tissue. To investigate the feasibility of atlasing studies, we applied PIP-seq to samples derived from healthy breast tissue. In tandem, we performed scRNA-seq on tissues from the same individuals using a commercially available scRNA-seq technology (10x Genomics, Chromium v3). We integrated PIP-seq data across participants and recovered expected cell types by dimensionality reduction, including the two lineages of luminal epithelial cells (LEP1 and LEP2), myoepithelial cells, fibroblasts, vascular cells and immune cells (Fig. 3a and Extended Data Fig. 3a,b)^31^. To compare transcriptome capture between platforms, we downsampled the 10x Chromium and PIP-seq datasets to an equivalent number of cells and reads (2,400 cells and 36,500 reads per cell). Chromium detected more unique genes (2,298 versus 1,757, median) and transcripts (7,491 versus 3,394) per cell, with similar percentages of reads assigned to mitochondrial transcripts (2.34% versus 1.32%; Extended Data Fig. 3c). To compare the transcriptome accuracy of PIP-seq, we downsampled each dataset to an equivalent number of UMIs per cell (2,400 cells and 1,500 UMIs), integrated the data, performed dimensionality reduction and identified clusters (Fig. 3b,c). We compared marker genes and the correlation between gene expression profiles by cluster. Predicted marker genes were concordant between methods (Fig. 3d), gene expression was highly correlated (Fig. 3e and Extended Data Fig. 4a), and breast tissue markers from previous reports were segregated identically within integrated clusters (Extended Data Fig. 4b). Comparison of PIP-seq to publicly available data from 10x (v3 and v2) and previously published scRNA-seq workflows demonstrated that PIP-seq produced high-quality transcriptomes across a range of sequencing depths (Extended Data Fig. 5). Next, we validated the scalability of PIP-seq, capturing and performing scRNA-seq on 138,146 breast tissue cells in a single-tube reaction and on 65,000 peripheral blood mononuclear cells (PBMCs; Extended Data Fig. 6a–c). At high cell numbers, we identified a population of CD34^+^ hematopoietic stem/progenitor cells in the PBMC sample, highlighting the importance of scalability in detecting rare cell types (Extended Data Fig. 6b,c). Last, we validated that PIP-seq is compatible with antibody-based cell hashing (Extended Data Fig. 6d,e). Hashing can be used to further increase the number of cells and conditions processed. Thus, PIP-seq is an easy-to-use, accurate and scalable method to profile complex tissues.

### PIP-seq for single-cell pooled CRISPR screens

**Fig. 4:**
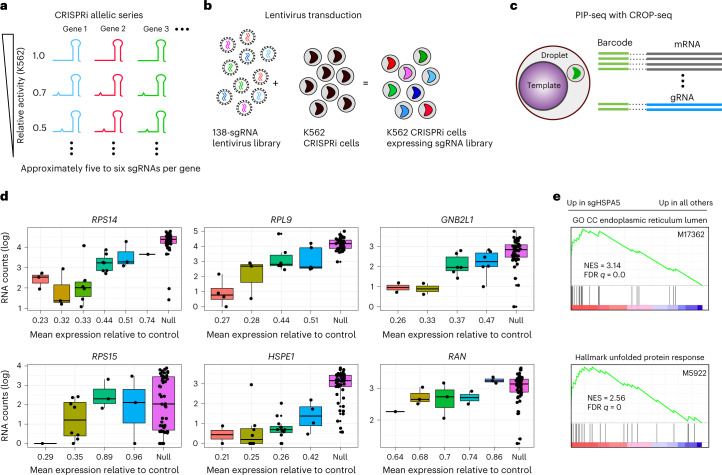
Transcriptome and gRNA sequencing using PIP-seq. **a**, Schematic of the CROP-seq sgRNA library designed with target mismatches to modulate the activity of essential genes. **b**, Lentiviral transduction of the CRISPRi library in K562 cells. **c**, Schematic of the capture and barcoding of polyadenylated mRNA and sgRNA using PIP-seq. RNA and sgRNA libraries are prepared separately and pooled for sequencing. **d**, Quantification of gene expression of sgRNAs within an allelic series. sgRNAs are ordered from high to low predicted knockdown efficiency^35^. Non-targeting sgRNAs are denoted as “Null”. Box plots indicate the median, with the lower and upper hinges corresponding to the 25th and 75th percentiles, respectively, and raw data points are displayed (with slight jitter). **e**, Preranked gene set enrichment analysis (GSEA) of scRNA-seq data comparing sgHSPA5-transduced cells to non-sgHSPA5-transduced cells shows enrichment in genes related to endoplasmic reticulum stress and unfolded protein response; GO CC, Gene Ontology cellular component; NES, normalized enrichment score; FDR, false discovery rate.

**Extended Data Fig. 7:**
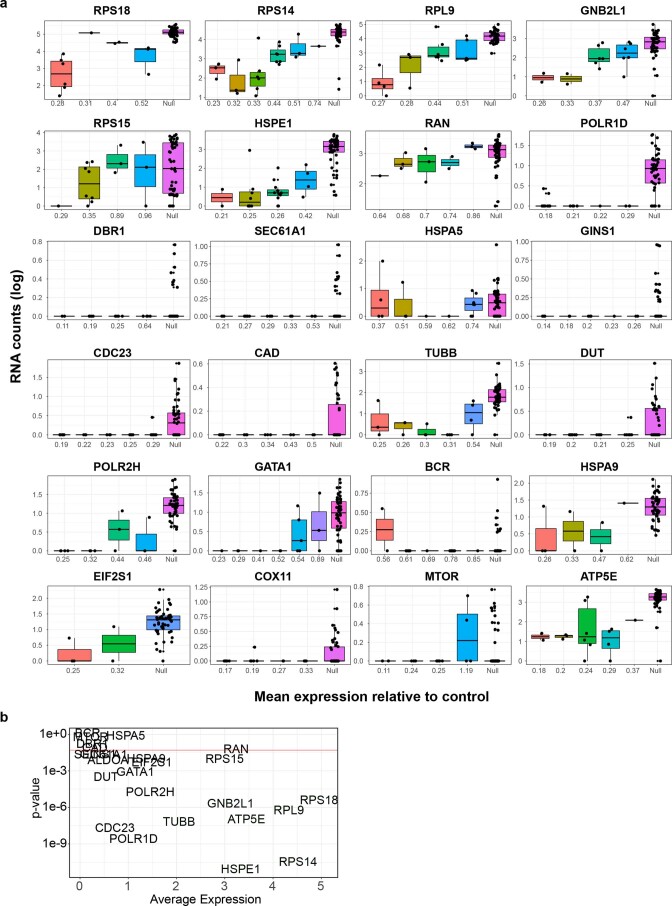
CROP-seq with PIP-seq. **(a)** Gene expression for each sgRNA within an allelic series for all genes in the CRISPRi library. Each sgRNA is ordered from predicted high to low knockdown efficiency. Non-targeting sgRNA are denoted as “Null.” Data is from one CROP-seq experiment. Box plots indicate the median with the lower and upper hinges corresponding to the 25th and 75th percentiles. Raw data points are displayed with a slight jitter. **(b)** The relationship between gene expression and predicted knockdown of each gene. Expected changes in transcription across the allelic series were prominent in highly expressed genes. p-value represents the significance of the generalized additive model relating gRNA identity to knockdown efficiency for each gene. P-values for each model were directly plotted along with the average expression for each gene (using log1p of the normalized counts). The horizontal red line shows the significance level of p = 0.05.

CRISPR perturbations combined with single-cell sequencing allow unbiased discovery of genotype–phenotype relationships^32–34^. Expanding this approach to genome-wide sgRNA libraries can elucidate gene function on an unprecedented scale. However, such studies require sequencing millions of cells to characterize all perturbations in libraries with tens or hundreds of thousands of individual sgRNAs^19^. To demonstrate how the throughput of PIP-seq enables perturbation studies at scale, we profiled the transcriptional changes associated with a CRISPR interference (CRISPRi) allelic series CROP-seq library^35^. This library expressed sgRNA and a polyadenylated copy of the guide sequence from separate promoters. gRNAs were captured and barcoded with the cell’s polyadenylated mRNA, making this approach immediately compatible with PIP-seq. The library is designed to quantitatively titrate gene expression using sgRNAs with target site mismatches^35^, allowing us to compare measured gene expression to expected knockdown efficiency across each gene’s allelic series (Fig. 4a). We transduced K562 cells containing a stable dCas9-KRAB with the CRISPRi lentiviral library and performed PIP-seq to capture the transcriptional profiles and sgRNA identity of individual cells (Fig. 4b,c). For cells with single gRNA assignments, previously reported knockdown efficiencies^35^ correlated with the normalized counts of targeted genes (Fig. 4d) and were most significant for highly expressed genes (Extended Data Fig. 7a,b). In addition, the knockdown of genes produced known transcriptional changes. For example, gRNA targeting *HSPA5* resulted in endoplasmic reticulum stress and increased the unfolded protein response (Fig. 4e). These results validate the use of PIP-seq for CROP-seq experiments, paving the way for routine million-cell experiments that map genotype–phenotype relationships at the genome scale.

### Transcriptomic signatures of MPAL relapse

**Fig. 5:**
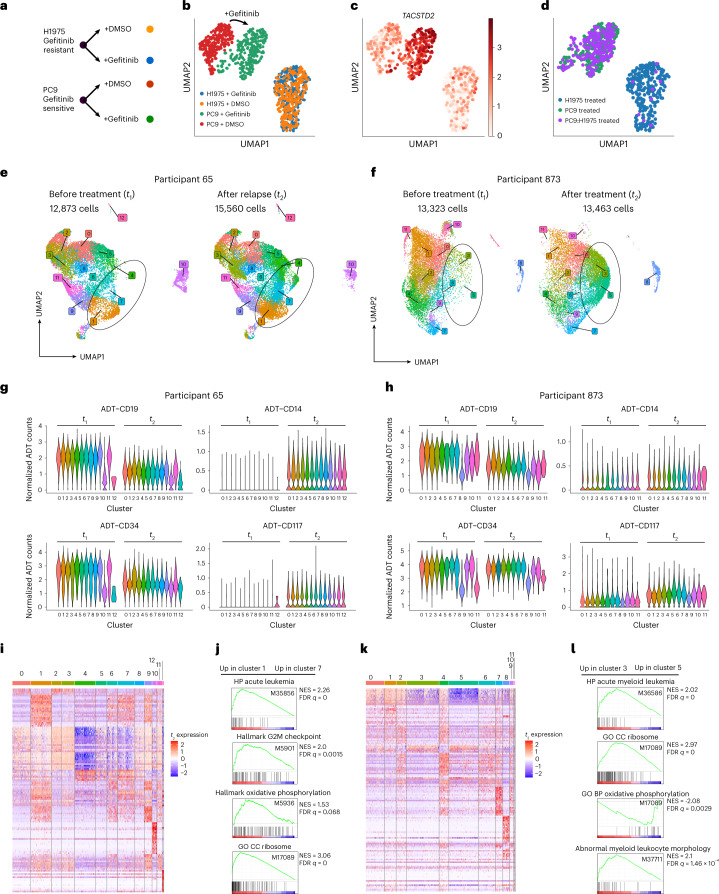
Molecular signatures of drug-resistant cancer phenotypes in cell lines and human samples. **a**, A two-by-two experimental study design using lung adenocarcinoma cell lines (H1975 and PC9) treated with gefitinib or DMSO. **b**, Clustering of scRNA-seq data after drug treatment shows transcriptional perturbations in gefitinib-sensitive PC9, but not gefitinib-resistant H1975, cells. **c**, Increased expression of *TACSTD2* in PC9 cells challenged with gefitinib. **d**, Identification of drug-resistant H1975 cells spiked into drug-sensitive PC9 cells based on gefitinib-induced transcriptional perturbation. **e**–**l**, PIP-seq RNA and barcoded antibody (CITE-seq) analysis of MPAL. **e**, Clustering of single cells for participant 65 before (left) and after (right) chemotherapy. **f**, Clustering of single cells for participant 873 before (left) and after (right) chemotherapy. **g**,**h**, ADT abundance, by cluster, before (*t*_1_) and after (*t*_2_) chemotherapy. ADTs change as a function of chemotherapy but are consistent among clusters for both participant 65 (**g**) and participant 873 (**h**), with the exception of T cell subsets. **i**–**l**, Analysis of transcriptional heterogeneity in MPAL samples. **i**, Heat map of top differentially expressed marker genes by cluster after relapse in participant 63. **j**, GSEA preranked analysis comparing transcriptomic differences between clusters 1 and 7 in participant 65 using the following gene sets: Human Phenotype acute leukemia (M35856), hallmark G2M checkpoint (M5901), hallmark oxidative phosphorylation (M5936) and Gene Ontology cellular component (GO CC) ribosome (M17089). **k**, Heat map of top differentially expressed marker genes by cluster after relapse in participant 873. **l**, GSEA preranked analysis comparing transcriptomic differences between clusters 3 and 5 in participant 873 using gene sets Human Phenotype acute myeloid leukemia (M36586), Gene Ontology cellular component ribosome (M17089), Gene Ontology biological process (GO BP) oxidative phosphorylation (M17089) and abnormal myeloid leukocyte morphology (M37711).

**Extended Data Fig. 8:**
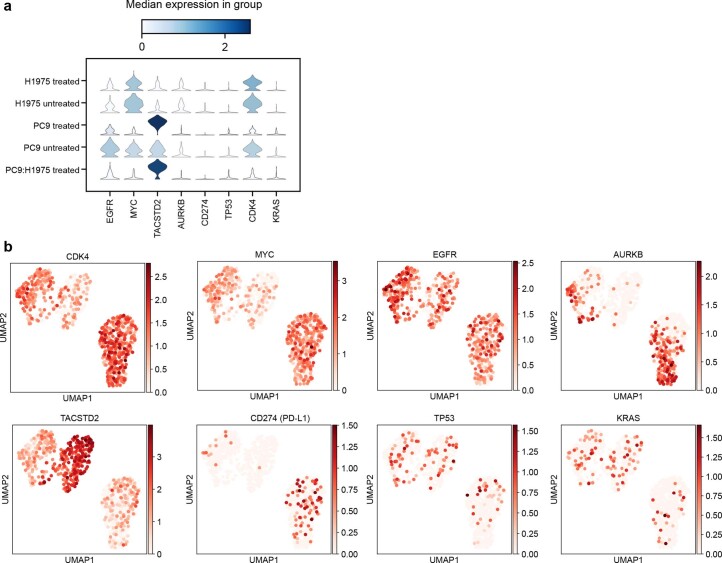
Identification of Gefitinib-specific transcriptional responses in cancer cell lines. **(a)** Violin plots of median expression values for selected differentially expressed genes. **(b)** The expression of selected differentially expressed genes superimposed on H1975 and PC9 cell clusters.

Monitoring of cancer in response to therapy is an emerging application of single-cell sequencing that benefits from rapid sample processing at the point of collection and the ability to delay cDNA synthesis and library preparation until multiple samples have been collected. We investigated the utility of PIP-seq for understanding cancer dynamics by first validating the single-cell transcriptional responses of two cancer cell lines (H1975 and PC9) to gefitinib, an epidermal growth factor receptor (EGFR) tyrosine kinase inhibitor. We treated H1975 and PC9 cells with DMSO (vehicle control) or 1 μM gefitinib overnight and performed PIP-seq (Fig. 5a). A transcriptional response in H1975, which is resistant to gefitinib due to EGFR mutations L858R and T790M, was not observed, while gefitinib-sensitive PC9 cells showed a substantial shift in gene expression (Fig. 5b). Differential gene expression analysis revealed increased levels of tumor-associated calcium signal transducer 2 (*TACSTD2*) in PC9 cells, consistent with its known modulation during lung adenocarcinoma tumor growth^36^ (Fig. 5c), and decreased expression of cyclin dependent kinase 4 (*CDK4*), which is known to enhance sensitivity to EGFR inhibitors^37^ (Extended Data Fig. 8). In addition, drug-resistant H1975 cells spiked into a background of sensitive cells (1:9 H1975:PC9) could be detected solely by their single-cell phenotypes and at roughly the expected frequency (4.7%; Fig. 5d). Thus, PIP-seq recovered genes with reported roles in lung cancer drug resistance and could identify resistance phenotypes within a background of drug-sensitive cells.

**Extended Data Fig. 9:**
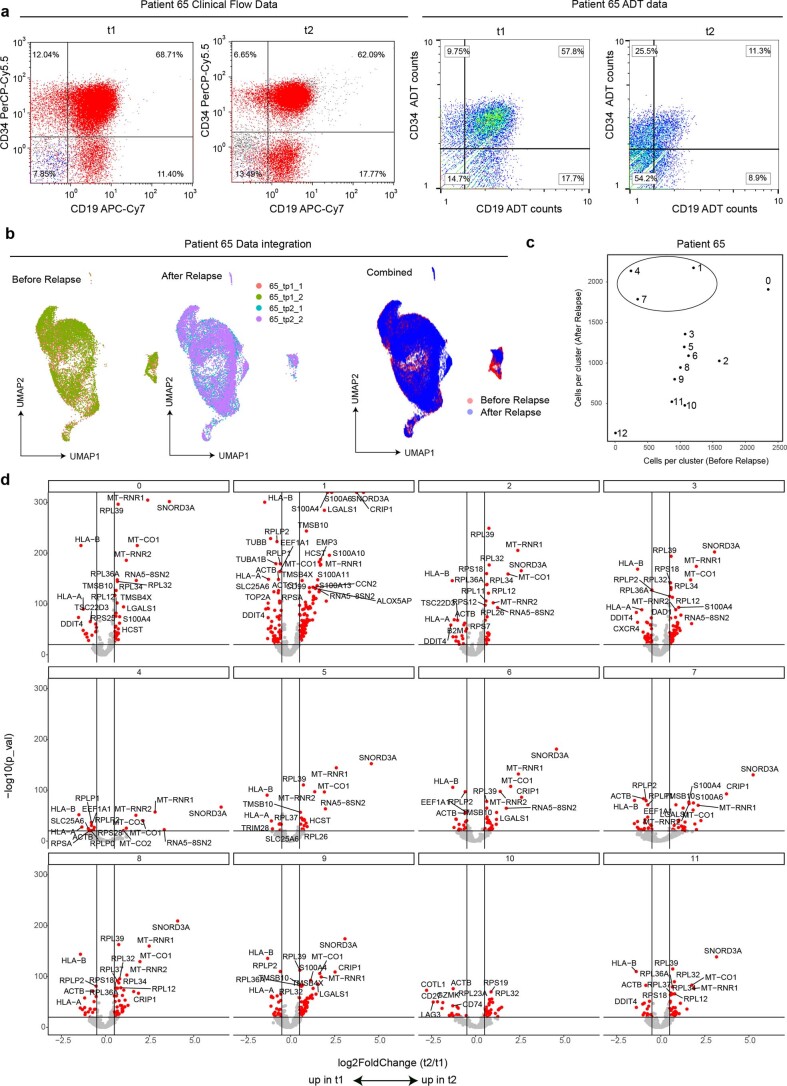
Analysis of PIP-seq data from MPAL Patient 65. **(a)** Clinical flow cytometry and corresponding antibody derived tag (ADT) data for patient 65 with mixed phenotypical acute leukemia (MPAL). **(b)** Integration of replicates and time points. **(c)** Correlation between the number of cells in each cluster before and after relapse identifies expansion of clusters 1,4, and7. **(d)** Volcano plots showing differential gene expression, by cluster, between t1 (before treatment) and t2 (after relapse).

**Extended Data Fig. 10:**
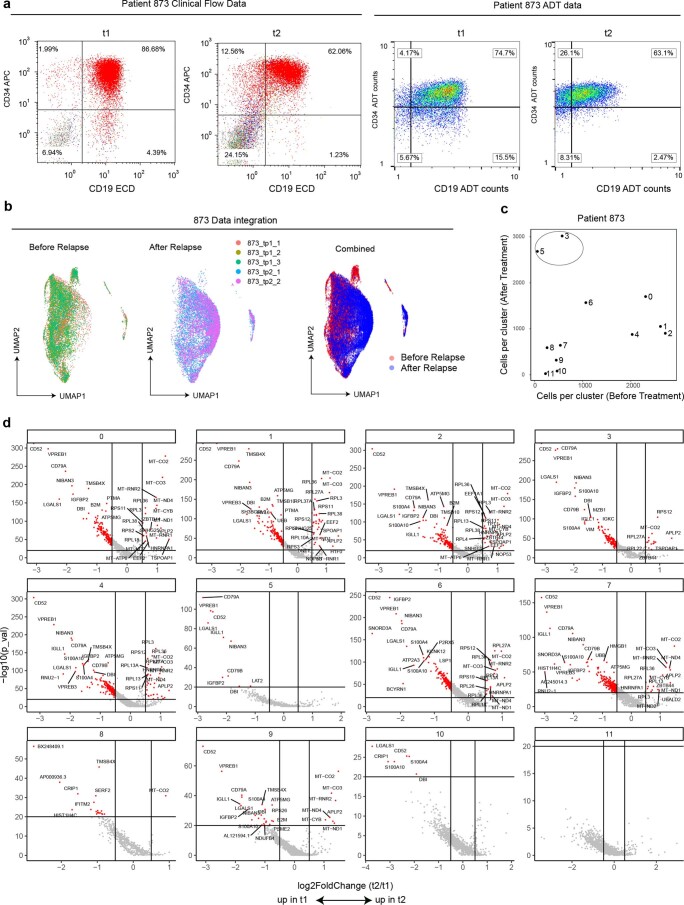
Analysis of PIP-seq data from MPAL Patient 873. (**a)** Clinical flow cytometry and corresponding antibody derived tag (ADT) data for patient 873 with mixed phenotypical acute leukemia (MPAL). **(b)** Integration of replicates and time points. **(c)** Correlation between the number of cells in each cluster before and after treatment identifies the expansion of clusters 3 and 5. **(d)** Volcano plots showing differential gene expression, by cluster, between t1 (before treatment) and t2 (after relapse).

Next, we applied PIP-seq to study MPAL, a high-risk disease characterized by multiple hematopoietic lineages^38,39^. Recurrence and changes in immunophenotype with chemotherapy are typically monitored using flow cytometry of surface markers during diagnosis, treatment and relapse, but this provides limited insight into the drivers of relapse after drug treatment. Like other scRNA-seq methods, PIP-seq can be multiplexed to simultaneously characterize single-cell gene expression and surface immunophenotype^40^. Using PIP-seq, we performed antibody-derived tag (ADT) sequencing (CITE-seq) on longitudinal samples collected from individuals with MPAL treated with chemotherapy. PIP-seq confirmed the diagnosis of these samples as B/myeloid MPAL and identified aberrant expression of immune and stem cell markers that matched with clinical immunophenotypes determined by flow cytometry (Supplementary Table 2 and Extended Data Figs. 9a and 10a). However, PIP-seq revealed an additional layer of complexity undetectable by traditional immunophenotyping. Dimensionality reduction identified cell clusters that emerged after drug treatment (Fig. 5e,f and Extended Data Figs. 9 and 10). These clusters had similar immunophenotypes (Fig. 5g,h) but contained notable transcriptional heterogeneity (Fig. 5i,k and Supplementary Tables 4 and 5). Cell populations upregulating genes and pathways (oxidative phosphorylation, G2M checkpoint modulation and ribosome biogenesis) implicated in a variety of cancers, including acute lymphoblastic leukemia^41–50^, but not previously linked to MPAL were observed (Fig. 5j,l). Taken together, our results highlight the value of single-cell methodologies for studying the heterogeneous response of cancer subpopulations to chemotherapy and the potential for the integration of simple and reliable scRNA-seq into clinical workflows.

## Discussion

Genomics has progressed rapidly to high-throughput, multimodal single-cell analysis^40,51–54^. Further improvements in data quality, the ability to measure additional cellular properties and new computational approaches for understanding and integrating single-cell information^55–57^ will continue to refine our understanding of cell states. At the same time, there remains an unmet need for simplified workflows that scale in cell number and sample size and that allow for breaks in processing after initial sample collection. PIP-seq is a microfluidics-free scRNA-seq method that produces high-quality data using a simplified emulsification technique. Like other high-throughput single-cell approaches, PIP-seq is fundamentally a strategy to barcode mRNA from cells so that material can be pooled and sequenced. The core advantage of PIP-seq is the speed and simplicity of sample processing. Particle-templated emulsification forms monodispersed bead-containing emulsions in minutes with a standard laboratory vortexer, removing the need for instrumentation located in core facilities or hours of multichannel pipetting to perform split-pool indexing in plates. This expands access to single-cell technologies in several ways. First, PIP-seq reduces the need for sample transport, enabling immediate processing by technicians without prior training and collection and banking of samples from remote locations, including field sites. Second, PIP-seq allows infectious samples that require special precautions to be processed at the point of collection or in the biosafety facilities where they are stored. More generally, rapid sample processing eliminates the need for fixatives and minimizes transcriptional perturbations and batch artifacts associated with processing many samples in series.

In addition to workflow simplicity, PIP-seq is intrinsically scalable, handling cell inputs over five orders of magnitude (10 to 10^6^), making it well suited for screening genome-wide Perturb-seq experiments and large cell atlas studies. While methods based on combinatorial indexing scale efficiently to large cell numbers, PIP-seq has a simpler workflow and is also compatible with high-throughput processing of samples in plates, allowing many conditions and replicates to be run simultaneously. This has implications for data quality and biological discovery in single-cell experiments because the detection of true positives and reduction in false positives in differential expression analysis is improved by incorporating replicates and statistical methods that account for biological variability^58^. Increased flexibility in the number of samples that can be processed also enables difficult experimental designs, such as dose–response curves, time-course studies, combinatorial perturbations, single-cell sequencing of organoids and large drug screens. In addition, because PIP-seq can directly emulsify in plates, it integrates with robotic fluid handling and therefore comprises a drop-in solution for single-cell readouts in high-throughput experiments in academia or industry.

We confirmed the accuracy of PIP-seq as a single-cell genomics tool by profiling heterogeneous tissue and directly comparing our results to a commercial scRNA-seq platform (10x Genomics). PIP-seq cell-type classification, marker identification and gene expression levels were tightly matched with 10x data but detected fewer genes per cell. We attribute these differences to the extensive optimization that the commercial platform has undergone and suspect that, like other single-cell techniques^3–6,12,25,59^, further improvements to PIP-seq molecular biology will increase sensitivity. In addition, because PIP-seq emulsions are functionally equivalent to those made with microfluidics, our approach is immediately compatible with emerging advances, including improvements to the molecular biology of myriad multiomic profiling methods developed for other droplet microfluidic barcoding systems^40,57,60^.

Finally, we demonstrated the utility of PIP-seq in processing clinical samples. In combination with barcoded antibodies, we profiled the relapse of MPAL after chemotherapy. MPAL is a subtype of leukemia characterized by poor prognosis^61^, lineage ambiguity, lack of consensus regarding therapy and considerable intratumoral genetic and immunophenotypic heterogeneity^62,63^. The molecular mechanisms underlying treatment resistance in this complex disease remain undefined. Changes in gene expression have been linked to prognosis and treatment resistance in multiple cancers. However, tumor heterogeneity makes it unlikely that bulk sequencing methods would identify strong gene signatures associated with resistance in clinical samples. Using PIP-seq of longitudinal samples from two individuals with MPAL with disease progression after initial therapy, we identified transcriptional heterogeneity beyond that observed by immunophenotype and speculate that this heterogeneity may play a role in MPAL treatment resistance. We observed upregulation of genes and pathways previously associated with acute lymphoblastic leukemia in several cell subsets that emerged after chemotherapy and modulation of ribosomal genes in both individuals. Control of translation has been previously implicated in many cancers^41–46,64^, including leukemia, but has not yet been linked to MPAL progression and drug resistance, suggesting that therapeutics targeting ribosomal biogenesis and/or protein translation may also have therapeutic potential in MPAL^65^. Our results motivate the use of single-cell technologies for understanding MPAL tumor heterogeneity and response to chemotherapy and suggest that the broad adoption of such technologies for monitoring cancer progression (and tailoring treatment) is within reach. In summary, scRNA-seq provides unparalleled insight into cell heterogeneity but remains underutilized in many settings. PIP-seq addresses this with a simple, rapid and scalable workflow that can be used by any lab containing standard molecular biology equipment.

## Methods

### PK-triggered cellular lysis and mRNA capture

Mammalian cells were stained with Calcein AM (Thermo Fisher, C3099) in 1 ml of PBS with 0.04% bovine serum albumin (BSA) according to manufacturer’s instructions. After 30 min of incubation at room temperature on a rotisserie incubator (Isotemp, Fisher Scientific), cell suspensions were quantified with a Luna-FL automated cell counter and diluted in 1× PBS with 0.04% BSA. Calcein-stained cells (1,500) in 5 µl of 1× PBS with 0.04% BSA were added to 35 µl of barcoded hydrogel templates with 29 U ml^–1^ PK (NEB, P8107S) and 70 mM DTT (Sigma, D9779) and mixed for 10 pipette strokes. Care was taken to avoid generating bubbles when mixing cells with barcoded hydrogel templates. Two hundred and eighty microliters of 0.5% ionic Krytox in HFE 7500 oil^66^ was added to the cell–bead mixture and vortexed at 3,000 r.p.m. for 15 s horizontally and then 2 min vertically with a custom vortexer (Fluent BioSciences, FB0002776). Oil was removed from below the emulsion such that less than 100 µl remained. The PIP emulsion was subsampled on a C-Chip disposable hemacytometer (Fisher Scientific, DHCN015) before lysis, with each subsample consisting of 3.5 µl of PIP emulsion per field of view. The C-chip was imaged in brightfield at ×2 magnification. The remaining PIP emulsion was subjected to enzymatic lysis at 65 °C for 35 min on a PCR thermocycler (Eppendorf Mastercycler Pro) with the lid temperature set to 105 °C. After lysis was complete, fluorescence images were captured using a Nikon 2000 microscope with 470-nm excitation (Thorlab, M470L5).

### Synthesis of barcoded bead templates

Prototype barcode bead fabrication proceeded according to previous reports^30^. Briefly, a simple coflow microfluidic device was used to combine acrylamide premix (6% (wt/vol) acrylamide, 0.1% bis-acrylimide, 0.3% (wt/vol) ammonium persulfate, 0.1× Tris-buffered saline–EDTA (TBSET: 10 mM Tris-HCl (pH 8.0), 137 mM NaCl, 20 mM EDTA, 1.4 mM KCl and 0.1% (vol/vol) Triton X-100), 50 µM acrydited primer (/5Acryd/TTTTTTTAAGCAGTGGTATCAACGCAGAGTACGACTCCTCTTTCCCTACACGACGCTCTTCC) with oil (HFE 7500, 3M Novec) containing 2% (wt/vol) surfactant (008-Fluoro-surfactant, Ran Technologies) and 0.4% (vol/vol) tetramethylethylenediamine). The emulsion was solidified at room temperature for 12 h, and beads were removed using 1*H*,1*H*,2*H*,2*H*-perfluoro-1-octanol (Sigma-Aldrich) and washed three times with Tris-EDTA-Tween buffer (TET: 10 mM Tris-HCl (pH 8.0), 10 mM EDTA and 0.1% (vol/vol) Tween 20), followed by two washes with 30 mM NaCl, 10 mM Tris-HCl (pH 8.0), 1 mM MgCl_2_ and 0.1% Tween 20. The final bead size was 80 µm. Split-pool barcode assembly used the ligation assembly approach as described previously^30^. Beads were resuspended in T4 ligation buffer (NEB, B0202S), heated with a complementary oligonucleotide to 75 °C for 2 min and cooled to room temperature to anneal. One hundred microliters of beads was distributed into each well of a 96-well plate containing a unique barcode with 1× T4 ligation buffer and 1.9 U µl^–1^ T4 DNA ligase (NEB, M0202M). Ligations were incubated at 25 °C for 1 h and heat inactivated at 65 °C for 10 min. Well contents were combined and washed five times in 15 ml of TET. The process was repeated to add four barcodes and a UMI with poly(T) (NNNNNNNNNNNNTTTTTTTTTTTTTTTTTTTV). Quality control steps were identical to previous reports^30^. Bead manufacturing methods were transferred to Fluent BioSciences for scaled production, validation and distribution. Commercially produced beads were used for several experiments, as noted.

### Varied format emulsification

PIP emulsification in varied formats was performed in 0.5-ml microcentrifuge tubes, 15-ml conical tubes and 50-ml conical tubes. Briefly, PIP particles were suspended in buffer with 29 U ml^–1^ PK (NEB, P8107S) and 70 mM DTT (Sigma, D9779) and pelleted through centrifugation. Barcoded hydrogel templates were then distributed at 35-µl, 0.5-ml and 8-ml volumes in 0.5-ml, 15-ml and 50-ml tubes, respectively. Fluorinated oil with surfactant (Fluent Biosciences, FB0001804) was added to each tube at 200-µl, 8-ml and 32-ml volumes, respectively. Emulsification was conducted on a Vortex Genie 2 with a custom adapter (Fluent, FBS-SCR-8VX) at maximum r.p.m. for 1 min. After emulsification, the samples were allowed to settle for 30 s, and excess oil was removed via syringes using 22-gauge blunt needles. The emulsion was subsampled, loaded on a C-Chip disposable hemacytometer (Fisher Scientific, DHCN015) and imaged under brightfield microscopy (DIAPHOT300, Nikon) at ×2 and ×4 magnification.

Emulsification in well plates was tested using two bead buffer conditions. First, to test emulsification in 96-, 384- and 1,536-well plates, PIP particles were suspended in 2% (vol/vol) Triton X-100 (Sigma, X100-5ML) in 10 mM Tris-HCl (Teknova, T1075) and centrifuged at 6,000*g*; the supernatant was then removed (Fig. 1 and Extended Data Fig. 2c). Depending on the well plate working volume, 38 µl, 8 µl or 3 µl of the centrifuged barcoded hydrogel templates was added to 96-, 384- or 1,536-well plates, respectively. For 96- and 384-well plates, 2 µl of sample was added to each well, and for 1,536-well plates, 1 µl was added to each well. PIP and sample volumes totaled 25% of the volume of each well. Each plate type was then sealed (Applied Biosystems, 4306311) and shaken for 5 min (IKA, 253614 and 3426400) to ensure complete mixing. Each plate type was centrifuged at 200*g* for 1 min before removing the seal. Then, 80 µl, 20 µl or 8 µl of 2% (wt/wt) fluorosurfactant (Ran BioTechnologies, 008 Fluorosurfactant) in HFE oil (3M, Novec 7500) was added to each well in 96-well (Applied Biosystems, N8010560), 384-well (Applied Biosystems, A36931) or 1,536-well (Nunc, 253614) plates, respectively. The addition of oil represented 50% of the volume of each well for a total volume of 75% consisting of PIP, sample and oil. After resealing, PIP emulsification was performed by vortexing for 30 s at 3,200 r.p.m. (Benchmark Scientific, BV1003). The emulsified plate was centrifuged at 200*g* for 1 min before removing the seal and imaging droplets from individual wells on a fluorescence microscope (EVOS FL Auto).

Second, to test well plate emulsification with cells in 96- and 384-well plates, PIP particles were suspended in buffer with 29 U ml^–1^ PK (NEB, P8107S) and 70 mM DTT (Sigma, D9779) and pelleted through centrifugation. For 96-well plates (Eppendorf, 0030129300), 25 µl of barcoded hydrogel templates was then distributed into each well with 4,000 cells per well (2,000 cells per µl × 2 µl). Fluorinated oil with surfactant (150 µl; Fluent Biosciences, FB0001804) was added to each well. Emulsification was conducted on a Vortex Genie 2 with a flat-head adapter at 3,000 r.p.m. for 2 min. For 384-well plates (Corning, 3347), 15 µl of barcoded hydrogel templates was then distributed into each well with 3,000 cells per well (2,000 cells per µl × 1.5 µl). Fluorinated oil with surfactant (105 µl; Fluent Biosciences, FB0001804) was added to each well. Emulsification was conducted on a Vortex Genie 2 with a flat-head adapter at 3,000 r.p.m. for 2 min (Fig. 1 and Extended Data Fig. 2a,b).

### PIP-seq protocol

Unless otherwise noted, cells were centrifuged at 300*g* for 5 min, washed twice in 1× PBS without calcium or magnesium (Thermo Fisher, 70011044) with 0.04% BSA, filtered with a 70-µm cell strainer and resuspended in 1× PBS with 1% Pluronic F127 (Sigma, P2443). Prealiquoted barcoded hydrogel templates were thawed on ice. Volumes of barcoded hydrogel templates, cells and oil varied based on the number of cells as noted in each experimental subsection below. The following protocol was used for a standard small-format run: 5 µl of 500 cells per µl was added to 35 µl of barcoded hydrogel templates with 29 U ml^–1^ PK and 70 mM DTT (Fluent BioSciences, FB0001876) and mixed for 10 strokes. Care was taken to avoid generating bubbles when mixing cells with barcoded hydrogel templates. Oil (280 µl; Fluent Biosciences, FB0001804) was added to the cell–bead mixture and vortexed (Vortex Genie 2, Scientific Industries) using a custom adapter (Fluent BioSciences, FB0002100) at the maximum r.p.m. for 15 s horizontally and 2 min vertically. Excess oil (230 µl) was removed, and the emulsion and enzymatic lysis was completed at 65 °C for 35 min with a 4 °C hold on a PCR thermocycler with the lid temperature set to 105 °C. The remaining oil was removed. The emulsion was broken using the following protocol. Using a multichannel pipette, 180 µl of room temperature high-salt buffer (250 mM Tris-HCl (pH 8), 375 mM KCl, 15 mM MgCl_2_ and 50 mM DTT) was added to the top of the emulsion followed by 40 µl of 100% 1*H*,1*H*,2*H*,2*H*-perfluoro-1-octanol (Sigma-Aldrich, 370533). The samples were vortexed for 3 s and briefly centrifuged, and the bottom oil phase was removed. Barcoded hydrogel templates were transferred into a 1.5-ml Eppendorf tube and washed three times with 2× RT buffer (100 mM Tris-HCl (pH 8.3), 150 mM KCl, 6 mM MgCl_2_ and 20 mM DTT) with 1% Pluronic F68 (Gibco, 24040032). After washing, the beads were pelleted, the aqueous layer was removed, and the remaining bead and buffer volume was 25 µl. To this bead buffer mixture, 25 µl of reverse transcription master mix comprising 4.8% PEG8000, 4% PM400, 2.5 µM template switch oligonucleotide (PIPS_TSO), 1 mM dNTPs (NEB), 1 U µl^–1^ RNase inhibitor (NxGen, Lucigen) and 1 U µl^–1^ reverse transcriptase (Thermo Fisher, Maxima H-minus EP0751) was added. The reaction was thoroughly mixed, and cDNA synthesis was completed for 30 min at 25 °C and 90 min at 42 °C, followed by 10 min at 85 °C and a 4 °C hold. Whole-transcriptome amplification (WTA) was performed directly on reverse transcription product without purification by adding 50 µl of 2× KAPA HiFi master mix and 0.25 µM primer (PIPS_WTA_primer) and thermocycling (95 °C for 3 min, 16 cycles of 98 °C for 15 s, 67 °C for 20 s and 68 °C for 4 min, followed by 72 °C for 5 min and a hold at 4 °C). After WTA, barcoded hydrogel templates were removed using Corning Spin-X filter columns (1 min at 13,000*g*), and amplified cDNA was purified using 0.6× Ampure XP. Libraries were generated from WTA amplified material using the Nextera XT DNA library preparation kit with a custom primer (PIPS_P5library) and standard Nextera P7 indexing primers (N70x). Libraries were pooled and sequenced using an Illumina NextSeq 2000 instrument with 15% PhiX. Oligonucleotides used in this study are supplied in Supplementary Table 1.

### Human–mouse mixing studies

Human HEK 293T cells (ATCC, CRL-3216) were grown in DMEM (Thermo Fisher, 11995073) supplemented with 10% fetal bovine serum (FBS; Thermo Fisher, A3840001) and 1% penicillin–streptomycin–glutamine (Thermo Fisher, 10378016). Mouse NIH 3T3 cells (ATCC, CRL-1658) were grown in DMEM (Thermo Fisher, 11995073) supplemented with 10% bovine calf serum (ATCC, 30-2030) and 1% penicillin–streptomycin–glutamine. Cells were grown to a confluence of ~70% and treated with TrypLE Express with Phenol red (Thermo Fisher, 12605010) for 3 min, quenched with an equal volume of growth medium and centrifuged for 5 min at 200*g*. The supernatant was removed, and the cells were resuspended in 1× DPBS without calcium or magnesium. Cells were diluted to their final concentration in 1× DPBS with 0.04% BSA and mixed evenly to create a 50:50 human:mouse mixture. Cell viability was evaluated using acridine orange/propidium iodide stain (Logos Bio, F23001) and quantified with a Luna-FL automated cell counter. Cells were processed using the PIP-seq protocol as described above.

### Seventy-two-hour hold experiments

Five microliters of a 50:50 mixture of human HEK 293T cells and mouse NIH 3T3 cells (800 cells per µl) was added to 35 µl of barcoded hydrogel templates (Fluent BioSciences, FB0003067) with 29 U ml^–1^ PK and 70 mM DTT and mixed for 10 strokes. Oil (280 µl; Fluent Biosciences, FB0001804) was added to the cell–bead mixture, which was vortexed on a digital vortexer using a custom adapter (Fluent BioSciences, FB0002084) at 3,000 r.p.m. for 15 s horizontally and 2 min vertically. Excess oil (230 µl) was removed, and the emulsion was placed in a preheated digital dry bath at 66 °C for 38 min and 4 °C for 11 min. Control samples proceeded to emulsion breaking, while 0 °C hold samples were placed in an ice bucket in the refrigerator (4 °C) for 72 h before breaking emulsions. Breaking, mRNA extraction, reverse transcription, WTA and cDNA isolation, adapter ligation-based library preparation and Illumina sequencing were performed as previously described.

### Healthy breast tissue comparison to 10x data

Fresh reduction mammoplasty tissue was processed as previously described^31,67^. Use of breast tissue specimens to conduct the studies described was approved by the University of California San Francisco Committee on Human Research under Institutional Review Board protocols 16-18865 and 10-01532. Tissues were obtained as deidentified samples, and all participants provided written informed consent. Bulk mammary tissues were mechanically processed into a slurry and digested overnight with collagenase type 3 (200 U ml^–1^, Worthington Biochem CLS-3) and hyaluronidase (100 U ml^–1^; Sigma-Aldrich, H3506) in medium containing charcoal:dextran-stripped FBS (GeminiBio, 100-119). The digested fragments were size filtered into a below-40-μm fraction and an above-100-μm fraction and cryopreserved. For PIP-seq, cells were thawed and resuspended in PBS + 0.04% BSA and passed through a 70-μm FlowMi cell strainer (Sigma, BAH136800070). For 10x Genomics data, the 100-μm fraction was thawed and further digested with trypsin, followed by dispase (Stemcell Technologies, 07913) and DNaseI (Stemcell Technologies, 07469) digestion to achieve single-cell suspensions. For PIP-seq, 20 µl of cells (1,500 cells per µl in PBS + 0.04% BSA) was added to 200 µl of barcoded hydrogel templates (Fluent BioSciences, FB0002617) and mixed for 10 strokes. Oil (1,000 µl; Fluent Biosciences, FB0001804) was added to the cell–bead mixture and vortexed on a digital vortexer using a custom adapter (Fluent BioSciences, FB0002100) at 3,000 r.p.m. for 15 s horizontally and 2 min vertically. Excess oil (800 µl) was removed, and the emulsion was placed on a preheated digital dry bath at 66 °C for 38 min and 4 °C for 11 min. Breaking, mRNA extraction, reverse transcription, WTA and cDNA isolation were performed under standard conditions. Adapter ligation-based library preparation was performed according to manufacturer’s instructions (Watchmaker Genomics, 7K0019-024). Samples were sequenced on an Illumina NextSeq 2000, with four participant samples pooled per P3 cartridge, and sequenced at a read depth of approximately 36,500 reads per cell. For 10x Genomics, cells from each participant were labeled with MULTIseq barcodes^13^ and were pooled and stained with DAPI to be sorted for DAPI-live cells. Single-cell libraries were prepared according to the 10x Genomics Single Cell V3 protocol (v3.1 Rev D) with the standard MULTIseq sample multiplexing protocol. The libraries were sequenced on a NovaSeq S4 lane at a read depth of about 70,000 reads per cell. To compare platforms, we downsampled PIP-seq and 10x data, which had different numbers of cells and sequencing depth per cell. The PIP-seq data had 54,825 cells, sequenced at approximately 36,500 reads per cell, while the 10x data had 2,420 cells sequenced at approximately 70,000 reads per cell. Data were downsampled to 2,400 cells and 36,500 reads in R (downsampleReads, DropletUtils). For correlation and marker gene comparisons, data were downsampled to 2,400 cells and 1,500 UMIs in R (SampleUMI, Seurat v4.1.0). Markers used for breast tissue cluster cell-type calling are available in Supplementary Table 2.

### Single-tube large-format breast tissue study

PIP-seq was performed as previously described, except that cells were counted and diluted with PBS + 0.04% BSA to a concentration of 10,000 cells per µl. Cell suspension (40 µl) was added to 800 µl of barcoded hydrogel templates (Fluent BioSciences, FB0003067). Oil (4,000 µl; Fluent Biosciences, FB0001804) was added to the cell–bead mixture and vortexed on a digital vortexer using a custom adapter (Fluent BioSciences, FB0002659) at 3,000 r.p.m. for 15 s horizontally and 2 min vertically. Excess oil was removed using a 3-ml syringe with a 22-gauge blunt-bottom syringe needle. Lysis proceeded using 3,300 µl of a lysis emulsion (Fluent BioSciences, FB0003039) added to the cell–bead emulsion. The mixture was placed in a preheated digital dry bath at 37 °C for 45 min and 4 °C for 10 min. Breaking, mRNA extraction, reverse transcription, WTA and cDNA isolation were performed under the same conditions as described previously. Adapter ligation-based library preparation was performed according to manufacturer’s instructions (Watchmaker Genomics, 7K0019-024). cDNA (80 ng) was used to prepare four replicate library preparations, which were pooled and sequenced on two Illumina NextSeq 2000 P3 cartridges at a read depth of 13,025 reads per cell after concatenation.

### CROP-seq

K562 CRISPRi cells were cultured in RPMI-1640 (Gibco, 11875093) with 10% FBS (Thermo Fisher Scientific, 10438026) and 1% penicillin–streptomycin (Thermo Fisher Scientific, 15140148) in an incubator at 37 °C with 5% CO_2_. K562 CRISPRi cells were transduced with a lentivirus library containing 138 sgRNAs^35^ at a multiplicity of infection of 0.1. Lentivirus-infected cells (BFP^+^) were sorted to high purity using a BD FACS Aria III (100-µm nozzle) and processed according to the PIP-seq scRNA-seq workflow. Cells (3 µl; 333 cells per µl) were added to 28 µl of barcoded hydrogel templates with 29 U ml^–1^ PK and 70 mM DTT and mixed for 10 strokes. One hundred and fifty microliters of 0.5% ionic Krytox in HFE 7500 oil was added to the cell–bead mixture and vortexed at 3,000 r.p.m. for 1 min on a Vortex Genie 2 with a custom tube adapter. cDNA was processed according to the standard PIP-seq protocol to obtain sequence-ready libraries containing transcriptome information. To recover sgRNA sequences, we implemented an additional amplification step. We amplified 1 ng of cDNA in a 50-µl reaction using primers P5-PE1 (0.5 µM) and Weissman_U6 (0.25 µM; Supplementary Table 1) with 1× Kappa HiFi. Reactions were thermocycled at 95 °C for 3 min followed by 10 cycles of 95 °C for 20 s, 70 °C for 30 s (−0.2 °C per cycle) and 72 °C for 20 s, followed by 8 cycles of 95 °C for 20 s, 68 °C for 30 s and 72 °C for 20 s, followed by 72 °C for 4 min and hold at 4 °C. Library PCR product enriched in sgRNA sequences was purified with a double-sided 0.5×/0.8× Ampure XP bead cleanup, and the size was determined (Agilent Tapestation).

Transcriptome and sgRNA libraries were pooled at 20:1 before sequencing. Reads were first processed to extract sgRNA sequences. The bioinformatics pipeline was run using a custom index built from the full human transcriptome (GENCODE v32) and gRNA sequences (Salmon v1.2.0.). This approach led to the recovery of >14,000 unique gRNA counts across all cell-associated barcodes. Cells were assigned to gRNA groups using a previously reported approach^32^. Briefly, cells were classified as uniquely expressing a single gRNA species if the guide’s expression was at least tenfold higher than the sum of all other gRNAs. Similarly, cells were classified as containing multiple gRNAs in cases where the difference was smaller than 1. For the 581 single cells sequenced, 2 did not have any gRNA, 441 contained a single gRNA, and 138 contained multiple gRNAs. Cell barcodes were processed using Seurat v4.1.0. All gRNAs in the list of features were excluded from the identification of variable transcripts (feature selection) and in subsequent stages of dimensionality reduction and clustering. To understand the relationship between gRNAs and mRNA expression, gRNAs were ranked according to their expected level of knockdown, as reported previously^35^, and a generalized additive model was used to assess groupwise trends for each set of gRNAs.

### Lung adenocarcinoma cell line experiments

PC9 cells were obtained from the RIKEN Bio Resource Center (RCB4455). H1975 cells were obtained from ATCC (CRL-5908). Cells were cultured in RPMI-1640 (Gibco, 11875093) with 10% FBS, penicillin and streptomycin in an incubator at 37 °C with 5% CO_2_. Gefitinib (1 µM; Frontier Scientific, 501411677) or DMSO was added to culture flasks 24 h before cells were collected for processing. PC9 and H1975 cells were both treated with gefitinib and DMSO. To perform the cell mixing study, gefitinib-treated H1975 cells and gefitinib-treated PC9 cells were mixed at a ratio of 1:9 H1975:PC9. Five microliters of cells (400 cells per µl) was added to 28 µl of barcoded hydrogel templates with 22.8 U ml^–1^ PK and 28 mM DTT and mixed for 10 pipette strokes. One hundred and fifty microliters of 0.5% ionic Krytox in HFE 7500 oil^66^ was added to the cell–bead mixture and vortexed at 3,000 r.p.m. for 1 min on a Vortex Genie 2 with a custom tube adapter. Triplicate tubes of 400 cells were processed per treatment condition. Data were analyzed using Seurat v4.1.0.

### Healthy PBMCs

Cryopreserved PBMCs were obtained from a commercial provider (AllCells). Cells were thawed and prepared for PIP-seq as previously described in the MPAL study, except that the final cell dilution was made in 1× PBS + 0.04% BSA. For the high-cell-count PBMC study, PIP-seq was performed as previously described in the high-cell-number breast tissue study except that cells were counted and diluted with PBS + 0.04% BSA to a concentration of 4,300 cells per µl, and 44 µl of cell suspension was added to 800 µl of barcoded hydrogel templates (Fluent BioSciences, FB0003067). Cryopreserved PBMCs used for cell hashing were obtained from a commercial provider (AllCells) and prepared for PIP-seq as described previously. For the cell hashing study, cell staining and PIP-seq were performed according to the PIP-seq Single Cell Epitope Sequencing user guide (FB0002079). Briefly, 1 million PBMCs were resuspended in 47.5 µl of cell staining buffer (BioLegend, 420201), and 2.5 µl of TruStain FcX block (BioLegend, 422301) was added before mixing and incubating for 10 min on ice. Next, 1 µg of TotalSeqA antibody was diluted in cell staining buffer, and 50 µl of this antibody dilution was added to the blocked cells before incubation on ice for 30 min. Stained cells were washed in cell staining buffer three times and resuspended in 1× PBS + 0.04% BSA at 2,000 cells per µl. For PIP-seq, 20 µl of this cell resuspension was added to 200 µl of barcoded hydrogel templates (Fluent BioSciences, FB0002617) and processed through PIP-seq.

### MPAL

Participants whose samples were used in this study were treated at the University of California San Francisco. Samples were collected in accordance with the Declaration of Helsinki under Institutional Review Board-approved tissue banking protocols, and written informed consent was obtained from all participants. Sample clinical characteristics are available in Supplementary Table 3. Cryopreserved PBMCs were thawed by hand until approximately 85% of ice remained. Using a 5-ml serological pipette, 1 ml of 4 °C defrosting medium (DMEM with 20% FBS and 2 mM EDTA) was added dropwise to each sample, and, without disturbing the remaining ice pellet, the sample was carefully transferred dropwise to a preprepared 40-ml aliquot of 4 °C defrosting medium. This was repeated until the contents of the entire cryovial were transferred into the 50-ml conical of defrosting medium. The sample was inverted four to five times and centrifuged at 114*g* for 15 min at 4 °C with no brake. The supernatant was aspirated, and 10 ml of room temperature RPMI-1640 with 1% penicillin–streptomycin–glutamine was used to gently resuspend the cells. Cell clumps were manually removed, and, if necessary, cells were filtered through a 70-μm cell strainer into a fresh 50-ml conical. The sample was inverted two to three times and centrifuged at 114*g* for 10 min with low brake at room temperature. The supernatant was aspirated, and cells were resuspended in an appropriate volume of 1× PBS + 5% FBS. Cells were quantified with Acridine Orange (AO)/Propidium Iodide (PI), and viability was evaluated on the Luna-FL. One to 2 million cells were aliquoted into a new 15-ml conical tube and centrifuged at 350*g* for 4 min at 4 °C, the supernatant was aspirated, and the tube was placed on ice. Forty-five microliters of cold cell staining buffer (BioLegend, 420201) was added per 1 million cells and resuspended gently. Five microliters of Trustain FcX block (BioLegend, 422301) was added per 1 million cells and gently mixed 10 times with a wide-bore pipette tip. Cells were blocked on ice for 15 min. A custom pool of 19 TotalSeqA antibodies was obtained from BioLegend and diluted according to the manufacturer’s instructions. Immediately before use, antibodies were mixed and centrifuged at 10,000*g* for 4 min at 4 °C; 4.6 µl of 0.5 µg µl^–1^ antibody pool was added per 1 million blocked cells and gently mixed 10 times with a wide-bore pipette tip. The samples were incubated on ice for 60 min. Next, 3.5 ml of cold cell staining buffer was added, gently mixed with a wide-bore pipette tip and slowly inverted twice to mix. Cells were centrifuged at 350*g* for 4 min at 4 °C, and the supernatant was removed. The addition of cold cell staining buffer was repeated twice for a total of three washes. After the final supernatant aspiration, stained cells were resuspended in 1× PBS with 0.04% BSA and mixed five to ten times until cells were completely suspended without visible clumps. Cell concentration was determined with AO/PI, and viability was evaluated on a Luna-FL. Final dilutions were made in 1× PBS with 0.04% BSA. Twenty microliters of cells was added to 200 µl of barcoded hydrogel templates (1,000 cells per µl) and processed according to the PIP-seq Single Cell Epitope Sequencing user guide (FB0002079). Marker genes identified for participants 65 and 873 are available in Supplementary Tables 4 and 5, respectively. Clinical FACS data from participants 65 and 873 were analyzed with FlowJo.

### PIP-seq bioinformatic analysis

Analysis of sequencing data was performed using custom scripts to generate gene expression matrices starting from processed FASTQ sequences. The pipeline is composed of four basic steps: (1) barcode identification and error correction, (2) mapping to reference sequences, (3) cell calling and (4) gene expression matrix generation. Briefly, after demultiplexing the sequencing data, each read in the FASTQ is matched against a ‘whitelist’ of known barcodes. Reads were matched with a hamming distance tolerance of 1, meaning that the barcode portion of a read can differ from a whitelist entry by one base and can still be matched to that barcode. Reads that did not match any barcode in the whitelist were discarded from further analysis. Matching reads were output to a new intermediate FASTQ file that was then used for mapping against an appropriate transcriptome reference. Reference transcriptomes matching the species of each sample were prepared using the Salmon ‘index’ function with the default *k*-mer size of 31 (ref. ^68^). GENCODE references were used to build the transcriptome indexes, including GRCh38.p13 for human, GRCm38.p6 for mouse and the combination thereof for HEK 293T/NIH 3T3 cell mixture studies. Following barcoding, Salmon ‘alevin’ v1.2.0 (ref. ^69^) was used to map reads to the full transcriptome. The intermediate FASTQ files generated during barcoding were provided as input into alevin along with a list of all whitelisted barcodes contained in raw reads. After mapping, data were output as UMI count matrices (sparse matrix, gene list and barcode list) with dimensions of ‘all barcodes x all genes in index’. An in-house Python implementation of emptyDrops^70^, a standard scRNA-seq method to separate putative cells from background, was then applied. A custom threshold for each experiment was set, beneath which no true cell barcodes were expected to fall. As with emptyDrops, an estimated ambient profile across all barcodes beneath that threshold was created. A *P* value was computed by comparing the gene expression profile for each barcode above the threshold against the ambient profile. Barcodes with a statistically significant difference (Benjamini–Hochberg-adjusted *P* value of <0.001) from the ambient background profile were categorized as cell-containing barcodes. The alevin output matrices were then subset to only include called cell barcodes. Gene expression matrices were normalized before performing unsupervised clustering and uniform manifold approximation and projection (UMAP) dimensionality reduction. Gene expression counts for each cell were first divided by the total counts for that cell and multiplied by a scaling factor of 10,000. The data were then transformed to natural log scale using log1p(). The Seurat package (v4.1.0) was used to perform downstream clustering, marker gene determination and visualization in R. Seurat’s FindClusters() and RunUMAP() commands were used with default settings.

For saturation curve comparisons, PIP-seq and 10x samples were downsampled to matching depths of 5,000–80,000 reads per called cell. Downsampling was performed using seqtk for PIP-seq samples and using the DropletUtils read10xMolInfo() function with a molecule_info.h5 file directly downloaded from the 10x website. Inflection point-based cell calling was used to standardize cell calls across platforms. Median transcripts per cell and genes per cell values were calculated from the cell fraction of the resulting count matrices. For violin plot comparisons, samples were prepared to match the same processing configuration used by Ding et al.^28^. Samples were first downsampled to 53,000 reads per called cell and trimmed to 50 bp for read 2 before processing, sampling in the same manner described above. Each violin plot represents the cell fraction from a single replicate of an HEK 293T/NIH 3T3 cell mixture, with human and mouse split out into separate plots.

Analysis of PBMC data for the high-cell-count study was performed using custom scripts, as described above, until the completion of mapping. Cell calling, clustering and differential expression were performed using PIPseeker v1.0.0 (Fluent Biosciences) in ‘reanalyze’ mode using –force-cells 65000. The top differentially expressed genes from the PIPseeker graph-based clustering result were used to determine cell types by comparing to a reference gene list (Supplementary Table 7). The log-normalized expression values for key genes (for example, *CD34*) were overlaid on the UMAP projection to highlight markers associated with specific cell types (color bars are in log_10_ scale). Analysis of PBMC data for the cell hashing study was performed using PIPseeker v1.0.0 in ‘count’ mode using STAR (v2.7.10a) and the PIPseeker human reference (https://www.fluentbio.com/products/pipseeker-for-data-analysis/). ADT analysis was conducted by performing barcode error correction with PIPseeker v1.0.0 (count mode) and custom scripts to trim read two to the first 16 bp. Error-corrected and trimmed FASTQ files were input to CITE-seq Count (v1.4.3) using the following settings: -t (hashtag whitelist) -cbf 1 -cbl 16 -umif 17 -umil 28–cells (number of called cells from RNA cell calling). The hashtag whitelist contained two TotalSeqA anti-human antibody hashes (A0253, TTCCGCCTCTCTTTG; A0255, AAGTATCGTTTCGCA). The filtered matrix output by PIPseeker for the RNA data was merged with the UMI count matrix from CITE-seq Count on cell barcode to create a merged matrix. The hashing data were demultiplexed in Seurat using HTODemux (positive.quantile=0.99). Downstream analysis was performed in Seurat using SCTransform() along with RunPCA(), FindNeighbors(dims=1:15) and RunUMAP(dims=1:15). Cell-type annotation was performed with singleR (v1.4.1) and used an annotated 10x Genomics v1 chemistry dataset as a reference. Cells were classified by their max hash identity and projected in the RNA-based UMAP space. The hash tag oligonucleotide data were subjected to clustering in Seurat using the HTOHeatmap() function to visualize singlets, doublets and unclassified cells.

For 72-h hold experiments, analysis was performed using custom scripts, as previously described above. Samples were normalized to the same depth (45,000 reads per cell). Cell types were then annotated as human (HEK 293T) or mouse (NIH 3T3) using a purity threshold of >85% single-species content per barcode. Barcodes from each species were subset, and transcript counts were summed for each gene to generate two pseudobulk count tables per sample. Samples were aggregated separately for each species and analyzed with DESeq2. A contrast of 0 versus 72 h was performed for each species while controlling for batch effects associated with different users. For the correlation analysis, pseudobulk counts derived above were normalized to transcripts per million and transformed using log(1 + x). Pearson correlations (*R*) and slopes (*m*) were calculated by fitting a linear model to the data. Data were then plotted in R with ggplot2 v3.3.5 and were aggregated into a grid using GGally v2.1.2. Additionally, the distribution of cells in UMAP space at 0 and 72 h after lysis was examined. After processing data in Seurat, as described, harmony batch correction was used to integrate datasets.

### Reporting summary

Further information on research design is available in the Nature Portfolio Reporting Summary linked to this article.

## Online content

Any methods, additional references, Nature Portfolio reporting summaries, source data, extended data, supplementary information, acknowledgements, peer review information; details of author contributions and competing interests; and statements of data and code availability are available at 10.1038/s41587-023-01685-z.

### Supplementary information

Reporting Summary

Supplementary Table 1 Oligonucleotides used in this study.

Supplementary Table 2 Breast tissue marker genes used for cluster cell-type identification (**a**) and identified using differential expression between clusters (**b**; FindAllMarkers Seurat v4.1.0, only.pos=FALSE, min.pct = 0.25, logfc.threshold = 0.5, test.use=‘bimod’).

Supplementary Table 3 Clinical characteristics of study participants.

Supplementary Table 4 Marker gene clusters for participant 65 identified using combined *t*_1_ and *t*_2_ time points (FindAllMarkers Seurat v4.1.0, only.pos=TRUE, min.pct = 0.25, logfc.threshold = 0, test.use=‘bimod’).

Supplementary Table 5 Marker gene clusters for participant 873 identified using combined *t*_1_ and *t*_2_ time points (FindAllMarkers Seurat v4.1.0, only.pos=TRUE, min.pct = 0.25, logfc.threshold = 0, test.use=‘bimod’).

Supplementary Table 6 Sequencing metrics.

Supplementary Table 7 PBMC marker genes used for cluster-based cell-type identification (**a**) and identified using differential expression between clusters (**b**).

## Data Availability

Sequencing data were deposited into Gene Expression Omnibus SuperSeries accession number GSE202919.

## Code availability

Code for processing raw FASTQ reads into count tables and UMAPs is available at https://www.fluentbio.com/products/pipseeker-software-for-data-analysis/. All other code will be made available from the corresponding author upon request.
