# Supplementary material for: Microfluidics-free single-cell genomics with templated emulsification
Source: Nat Biotechnol. 2023 Mar 6;41(11):1557–66. doi: 10.1038/s41587-023-01685-z (PMC10635830; doi:10.1038/s41587-023-01685-z)
Supplement: Supplementary file 1 — Reporting Summary [file 41587_2023_1685_MOESM1_ESM.pdf]

nature research

Corresponding author(s): Adam Abate

Last updated by author(s): Jan 13, 2023

Reporting Summary

Nature Research wishes to improve the reproducibility of the work that we publish. This form provides structure for consistency and transparency in reporting. For further information on Nature Research policies, see our Editorial Policies and the Editorial Policy Checklist.

Statistics

For all statistical analyses, confirm that the following items are present in the figure legend, table legend, main text, or Methods section.

n/a	Confirmed	
☐	☒	The exact sample size (*n*) for each experimental group/condition, given as a discrete number and unit of measurement
☐	☒	A statement on whether measurements were taken from distinct samples or whether the same sample was measured repeatedly
☐	☒	The statistical test(s) used AND whether they are one- or two-sided<br>*Only common tests should be described solely by name; describe more complex techniques in the Methods section.*
☐	☒	A description of all covariates tested
☐	☒	A description of any assumptions or corrections, such as tests of normality and adjustment for multiple comparisons
☐	☒	A full description of the statistical parameters including central tendency (e.g. means) or other basic estimates (e.g. regression coefficient) AND variation (e.g. standard deviation) or associated estimates of uncertainty (e.g. confidence intervals)
☐	☒	For null hypothesis testing, the test statistic (e.g. F, t, r) with confidence intervals, effect sizes, degrees of freedom and P value noted<br>*Give P values as exact values whenever suitable.*
☒	☐	For Bayesian analysis, information on the choice of priors and Markov chain Monte Carlo settings
☒	☐	For hierarchical and complex designs, identification of the appropriate level for tests and full reporting of outcomes
☐	☒	Estimates of effect sizes (e.g. Cohen's *d*, Pearson's *r*), indicating how they were calculated

Our web collection on statistics for biologists contains articles on many of the points above.

Software and code

Policy information about availability of computer code

Data collection	BD FACS Aria III
Data analysis	R (4.1.2); Seurat (v. 4.1.0); dplyr (1.0.8); FlowJo (10.8.0); ggplot2 (v3.3.5); GSEA (v 4.2.1); Adobe Illustrator (26.1); scanpy (1.4.4); anndata (0.7.1); umap (0.3.10); numpy (1.18.1); scipy (1.4.1); pandas (1.0.3); scikit-learn (0.22.1); CITE-seq-Count (v1.4.3); STAR (v2.7.10a); PIPseeker (v1.0.0).

For manuscripts utilizing custom algorithms or software that are central to the research but not yet described in published literature, software must be made available to editors and reviewers. We strongly encourage code deposition in a community repository (e.g. GitHub). See the Nature Research guidelines for submitting code & software for further information.

Data

Policy information about availability of data

All manuscripts must include a data availability statement. This statement should provide the following information, where applicable:

- Accession codes, unique identifiers, or web links for publicly available datasets
- A list of figures that have associated raw data
- A description of any restrictions on data availability

Sequencing data were deposited into GEO SuperSeries accession number GSE202919. The data has been made public. All other materials will be made available upon request.

April 2020

Field-specific reporting

Please select the one below that is the best fit for your research. If you are not sure, read the appropriate sections before making your selection.

☒ Life sciences ☐ Behavioural & social sciences ☐ Ecological, evolutionary & environmental sciences

For a reference copy of the document with all sections, see nature.com/documents/nr-reporting-summary-flat.pdf

Life sciences study design

All studies must disclose on these points even when the disclosure is negative.

Sample size	Most experiments related to single cell sequencing. Single cell RNA-seq was performed on 500 to 50,000 cells, consistent with samples sizes used by others in the field (PMID: 26000488, PMID: 26000487)
Data exclusions	No data were excluded from the study.
Replication	The replication number of each experiment is provided in the manuscript. Each replication was successful.
Randomization	For in vitro drug experiments, samples were randomly allocated into treatment groups. No other samples were allocated into experimental groups because samples (tissue, blood) were processed directly without treatment.
Blinding	Blinding was not performed. In most experiments, experimenters were required to know the conditions of each well.

Reporting for specific materials, systems and methods

We require information from authors about some types of materials, experimental systems and methods used in many studies. Here, indicate whether each material, system or method listed is relevant to your study. If you are not sure if a list item applies to your research, read the appropriate section before selecting a response.

Materials & experimental systems

n/a	Involved in the study	
☐	☒	Antibodies
☐	☒	Eukaryotic cell lines
☒	☐	Palaeontology and archaeology
☒	☐	Animals and other organisms
☐	☒	Human research participants
☒	☐	Clinical data
☒	☐	Dual use research of concern

Methods

n/a	Involved in the study	
☒	☐	ChIP-seq
☐	☒	Flow cytometry
☒	☐	MRI-based neuroimaging

Antibodies

Antibodies used	CITE-seq Antibodies: ADT-CD14 (TotalSeq™-A0081 anti-human CD14 Antibody) #M5E2; ADT-CD64 (TotalSeq™-A0162 anti-human CD64 Antibody) #10.1; ADT-CD22 (TotalSeq™-A0393 anti-human CD22 Antibody) #S-HCL-1; ADT-CD10 (TotalSeq™-A0062 anti-human CD10 Antibody) #HI10a; ADT-CD13 (TotalSeq™-A0364 anti-human CD13 Antibody) #WM15; ADT-CD117 (TotalSeq™-A0061 anti-human CD117 (c-kit) Antibody) #104D2; ADT-CD5 (TotalSeq™-A0138 anti-human CD5 Antibody) #UCHT2; ADT-CD7 (TotalSeq™-A0066 anti-human CD7 Antibody) #CD7-6B7; ADT-CD56 (TotalSeq™-A0084 anti-human CD56 (NCAM) Recombinant Antibody) #QA17A16 ; ADT-HLA-DR (TotalSeq™-A0159 anti-human HLA-DR Antibody) #L243 ; ADT-CD11b (TotalSeq™-A0161 anti-human CD11b Antibody) #ICRF44 ; ADT-CD4 (TotalSeq™-A0922 anti-human CD4 Antibody) #OKT4 ; ADT-IgG1 (TotalSeq™-A0090 Mouse IgG1, κ isotype Ctrl Antibody) #MOPC-21; ADT-CD3 (TotalSeq™-A0049 anti-human CD3 Antibody) #SK7; ADT-CD19 (TotalSeq™-A0050 anti-human CD19 Antibody) #HIB19; ADT-CD30 (TotalSeq™-A0028 anti-human CD30 Antibody) #BY88; ADT-CD33 (TotalSeq™-A0052 anti-human CD33 Antibody) #P67.6; ADT-CD45 (TotalSeq™-A0391 anti-human CD45 Antibody) #HI30; ADT-CD34 (TotalSeq™-A0054 anti-human CD34 Antibody) #581. Sample HHMTB0065: CD34 PerCP-Cy5.5 – clone 8G12, BD Biosciences Cat No 347213; CD19 APC-Cy7 – clone SJ25C1, BD Biosciences Cat No 348804. Sample HMTB00873; CD34 APC – clone 581, Beckman Coulter Cat No IM2472U; CD19 ECD – clone J3-119, Beckman Coulter Cat No IM2708U. Hash Antibodies: TotalSeq™-A0253 anti-human Hashtag 3, BioLegend Cat. No. 394605, Lot No. B364258; TotalSeq™-A0255 anti-human Hashtag 5 Cat. No. 394609, Lot No. B366838.
Validation	All antibodies were commercial in origin and validated by the company. BioLegend (https://www.biolegend.com/nl-nl/reproducibility) purifies antibodies from cell line supernatant using affinity chromatography followed by validation using proper negative and positive controls in functional assays. This is relevant for all antibodies used for flow cytometry and Ab sequencing. BD (https://www.bdbiosciences.com/en-us/products/reagents/flow-cytometry-reagents/research-reagents/quality-and-reproducibility) confirms specificity using multiple methodologies that may include a combination of flow cytometry, immunofluorescence, immunohistochemistry or western blot. Beckman Coulter (https://www.beckman.com/reagents/coulter-flow-cytometry/antibodies-and-kits/single-color-antibodies/quality-standards).

Eukaryotic cell lines

Policy information about cell lines

Cell line source(s)

Human HEK 293T cells (ATCC # CRL-3216), Murine NIH/3T3 cells (ATCC # CRL-1658) , H1975 (ATCC#CRL-5908); K562i were a gift from the Weissman lab; PC9 was obtained from RIKEN Bio Resource Center (RCB4455).

Authentication

Cell lines are authenticated by the manufacturer with STR profiling. K562i were generated and authenticated by the Weissman lab (PMID: 31932729)

Mycoplasma contamination

Cell lines are verified to be free of Mycoplasma contamination by the manufacturer.

Commonly misidentified lines
(See ICLAC register)

No commonly misidentified cell lines were used

Human research participants

Policy information about studies involving human research participants

Population characteristics

Breast tissue study: Samples were derived from a human breast tissue biobank at Univeristy of California in San Francisco (UCSF). This biobank was established from patients who underwent elective breast reduction or mastectomy surgeries at UCSF for cosmetic reasons. These patients are either cis-women or trans-men who are older than 18.

Cancer study: Two human participants were included in this study.Patient 65 is a 65 year old male diagnosed with B/myeloid mixed-phenotypic acute leukemia. This patient had an MLL rearrangement. Genotypic information (germline or somatic) was not obtained. The patient was treated with cytotoxic chemotherapy (cytarabine and duanorubicin).Patient 873 is a 76 year old female diagnosed with B/myeloid mixed-phenotypic acute leukemia. Genotypic information includes somatic mutations in TP53 (c.743G>A) and SF3B1 (c.1739C>T). The patient was treated with cytotoxic chemotherapy (cyclophosphamide and dexamethasone) and the antibody-drug conjugate inotuzumab.

Recruitment

Breast tissue study: Patients were selected from those who have already been scheduled for surgery, and those who are not excluded by being <18yrs of age or currently pregnant. Patients were notified by their physician during a clinical visit and the research team reached out to them before their surgeries to obtain consent.

Cancer study: Patients consented to have samples prospectively banked in the UCSF tumor bank at time of routine bone marrow biopsy. The samples were then retrospectively selected from the UCSF tumor bank for this project.

Self selection bias, should it exist, is not expected to impact the results of single cell RNA-seq studies that characterize cell heterogeneity.

Ethics oversight

Breast tissue study: This study uses human tissues previously collected in a biobank approved by UCSF IRB.

Cancer study: The prospective banking of patient samples is approved by the UCSF IRB (IRB# 11-06477). Samples were collected in accordance with the Declaration of Helsinki under institutional review board-approved tissue banking protocols, and written informed consent was obtained from all patients.

Note that full information on the approval of the study protocol must also be provided in the manuscript.

Flow Cytometry

Plots

Confirm that:

- ☒ The axis labels state the marker and fluorochrome used (e.g. CD4-FITC).
- ☒ The axis scales are clearly visible. Include numbers along axes only for bottom left plot of group (a 'group' is an analysis of identical markers).
- ☒ All plots are contour plots with outliers or pseudocolor plots.
- ☒ A numerical value for number of cells or percentage (with statistics) is provided.

Methodology

Sample preparation

Bone Marrow Aspirates (BMA) and Peripheral Blood Specimens are prepared using the Bulk Lysis Technique. Briefly, using a 3mL syringe with a 22 gauge blunt needle, bone marrow aspirate was drawn up and expelled back into the tube a minimum of three times to break up the spicules and release cells. Any clots in the sample were removed from the tube. Red blood cell lysis was performed using ammonium chloride. After tube inversion for 10 minutes, cells were centrifuges at 1400rpm for 5 minutes. Supernatant was removed, and the pellet was resuspended by adding, dropwise, 1mL of PBS + 0.1%NaN3 + 0.5% BSA to the tube while vortexing gently. An additional 13mL of PBS + 0.1%NaN3 + 0.5% BSA was added, clumps were removed manually with a pipette, and cells were centrifuges at 1400rpm for 5 minutes. Pellets were resuspended in 600µL of PBS-P solution (10% heat-inactivated (56°C for 30 min) newborn calf serum), with 10% Acid Citrate Dextrose (Sigma C3821-50ML).

Instrument	Sample HMTB0065: Instrument: BD FacsCanto (6-color)<br>Sample HMTB00873: Instrument: Beckman-Coulter Navios (10-color)
Software	Kaluza (Beckman Coulter)
Cell population abundance	Abundance is reported in figures where relevant.
Gating strategy	Sample HMTB0065: Gating strategy: none (all events displayed)<br>Sample HMTB00873: Gating strategy: none (all events displayed)

☒ Tick this box to confirm that a figure exemplifying the gating strategy is provided in the Supplementary Information.
