# Supplementary material for: Microfluidics-free single-cell genomics with templated emulsification
Source: Nat Biotechnol. 2023 Mar 6;41(11):1557–66. doi: 10.1038/s41587-023-01685-z (PMC10635830; doi:10.1038/s41587-023-01685-z)
Supplement: Supplementary file 4 — Clinical characteristics of study participants. [file 41587_2023_1685_MOESM4_ESM.pdf]

Patient ID	Age	Gender	Timepoint	MPAL Subtype	Clinical Immunophenotype	Therapy	Best Response
65	54	Male	Diagnosis	B/Myeloid	All blasts expressed CD34, CD38, HLA-DR, Majority subset: 82% of blast population expressed variable CD15, CD19, weak CD22, CD79a. Subset expressed TdT. Minority subset: 18% of blast population Expressed weak CD11c, weak CD13, CD33, CD64.	HDACi + Dauno	CR (MRD by flow cytometry 1%)
			Relapse	B/Myeloid	Expressed weak to absent CD15, CD19, CD38, Subset (69% of blast population) co-expressed CD34 and weak-to-absent CD22.		
873	76	Female	Diagnosis	B/Myeloid	Expressed weak CD4, CD13, CD19, weak CD22, CD34, CD38, weak CD71, weak CD123, HLA-DR, Subset co-expressed CD117 (21% of gated events positive). Subset co-expressed CD10 (11% of gated events positive).	miniCVD + Ino	None (MRD by flow cytometry 70%)
			Relapse	B/Myeloid	Expressed weak CD4, CD13, variable CD19, weak-absent CD22, CD33, bright CD34, weak CD38, weak-absent CD64, weak CD71, variable CD117, weak CD123, weak HLA-DR, Minor subset expressed possible CD10.		

CR = Complete Response; HDACi = histone deacetylase inhibitor; Dauno = daunorubicin; MiniCVD = cyclophosphamide and dexamethasone at 50% dose reduction. Ino.= inotuxumab ozogamicin; MRD = measurable residual disease
